# Enhancing water stress tolerance of bread wheat during seed germination and seedling emergence: caffeine-induced modulation of antioxidative defense mechanisms

**DOI:** 10.3389/fpls.2024.1336639

**Published:** 2024-06-27

**Authors:** Qasim Ali, Rashida Perveen, Farah Saeed, Hunaina Manzoor, Shafaqat Ali, Muhammad Iftikhar Hussain, Ajaz Ahmad

**Affiliations:** ^1^ Department of Botany, Government College University Faisalabad, Faisalabad, Pakistan; ^2^ Department of Physics, Government College for Women Ayub Research, Faisalabad, Pakistan; ^3^ Department of Botany, Government College Women University Faisalabad, Faisalabad, Pakistan; ^4^ Department of Environmental Sciences, Government College University Faisalabad, Faisalabad, Pakistan; ^5^ Department of Biological Sciences and Technology, China Medical University, Taichung, Taiwan; ^6^ Department of Plant Biology and Soil Science, Universidade de Vigo, Vigo, Spain; ^7^ Department of Clinical Pharmacy, College of Pharmacy, King Saud University, Riyadh, Saudi Arabia

**Keywords:** caffeine, seedling emergence, hydrolyzing enzymes, wheat, antioxidants, photosynthetic pigments

## Abstract

Better crop stand establishment, a function of rapid and uniform seedling emergence, depends on the activities of germination-related enzymes, which is problematic when there is insufficient soil moisture. Different ways are in practice for counteracting this problem, including seed priming with different chemicals, which are considered helpful in obtaining better crop stand establishment to some extent through improved seed germination and seedling emergence. In this growth room experiment, caffeine was used as a seed priming agent to improve germination under moisture scarcity. Polyethylene glycol-8000 (18%) was added to Hoagland’s nutrient solution to create drought stress (−0.65 MPa). The experiment was arranged in a completely randomized design (CRD), having four replications of each treatment. A newly developed wheat genotype SB-1 was used for the experimentation. Different doses of caffeine, i.e., 4 ppm, 8 ppm, 12 ppm, and 16 ppm, including no soaking and water soaking, were used as seed priming treatments. Water deficit caused oxidative stress and adversely affected the seed germination, seedling vigor, activities of germination enzymes, photosynthetic pigments, and antioxidative defense mechanism in roots and shoots of seedlings. Caffeine seed priming ameliorated the negative effects of water deficit on seed germination and seedling vigor, which was attributed to the reduction in lipid peroxidation and improvement in the activities of germination-related enzymes like glucosidase, amylase, and protease. Conclusively, seed priming with 12 ppm caffeine outperformed the other treatments and hence is recommended for better crop stand establishment under conditions of soil moisture deficit.

## 1 Introduction

Changing environmental conditions are severely disturbing the life cycles of plants in different ways, resulting in sever yield losses. Shortage of fresh water for irrigation and increase in evapotranspiration due to a rise in temperature along with changes in rainfall patterns are being considered the major constraints in this regard (Batool et al., 2022; Habib-ur-Rahman et al., 2022; Nyaupane et al., 2024). Semi-arid and arid areas, totally dependent upon rainfalls for their agricultural needs, are highly prone to such environmental variations. These areas contribute a major share of the world’s agricultural productivity. Moreover, the negative impacts of environmental variations are more pronounced in developing countries like Pakistan (Habib-ur-Rahman et al., 2022). Water deficiency negatively affects every stage of plant life, but seed germination and seedling establishment are considered the most vulnerable ones (Qayyum et al., 2011; Kizilgeçi et al., 2017; Song et al., 2019). Reduction in seed germination and seedling emergence leads to poor crop stand establishment with the ultimate decline in seed yield (Farajollahi et al., 2014; Muscolo et al., 2014; Kizilgeçi et al., 2017; Batool et al., 2022).

An optimum level of soil moisture is required for the proper germination of seeds, the deficiency of which critically hampers the process of germination and frequently depresses the seedling vigor (Basu et al., 2016) by disturbing the metabolic activities, necessary to start the seed germination and are also helpful for seedling development (Perveen et al., 2021). This depression in seed germination, under limited availability of moisture, is the function of reduction in the absorption of water by seed, necessary for imbibition (Hussain et al., 2018). The weak crop stand establishment, owing to poor germination and seedling emergence, is more problematic under rain-fed conditions in arid and semi-arid areas (Qayyum et al., 2011). It can only be overcome by improving and speeding up the processes of seed germination and seedling emergence (Saeed et al., 2023a) that sorely depends on the extent of water absorption by seed for imbibition, necessary for triggering the activities of germination-related enzymes, such as amylase (Amy), protease (Prot), and glucosidase (Gluco) (Ali et al., 2018). Better activities of these hydrolyzing enzymes catabolize the stored large bio-molecules into simpler ones, like fatty acids, amino acids, and sugars, which serve as raw materials for the developing seedlings (Perveen et al., 2021; Saeed et al., 2023a). These catabolic activities provide not only the basic simple molecules but also the energy to the developing seedlings (Perveen et al., 2021, Perveen et al., 2022; Saeed et al., 2023a).

To overcome the negative impacts of different stresses at any of the growth stages, different methods or techniques are being employed (Ali et al., 2019; Ali et al., 2020a, Ali et al., 2020b, Ali et al., 2020c; Perveen et al., 2021; Saeed et al., 2023a, Saeed et al., 2023b), including the screening of plant genotypes with improved germination potential (Ali et al., 2016, Ali et al., 2018), agronomic practices (Lamichhane and Soltani, 2020), and use of chemicals (organic and inorganic ones) through different ways at different growth stages (Ali et al., 2018; El Sabagh et al., 2019; Ali et al., 2021; Shehzad et al., 2022). Seed priming not only improves seed germination to obtain a good crop stand but also induces tolerance in plants to various stresses at later growth stages (Lutts et al., 2016; Noman et al., 2018a, Noman et al., 2018b; Nawaz et al., 2020; Ali et al., 2020a; Habib et al., 2021; Arun et al., 2022). It is considered a low-cost approach for obtaining better production by establishing good crop stands (Zulfiqar, 2021), not only under limited water supply but also in well-irrigated conditions (Ali et al., 2020a; Habib et al., 2021). Pre-sowing seed treatment is gaining the attention of researchers due to its low cost and significant positive outcomes at the global level (Noman et al., 2018a, Noman et al., 2018b; Ali et al., 2020a; Habib et al., 2021). Various types of seed priming agents are in practice, but the use of eco-friendly organic compounds, having a significant role in seed germination and seedling establishment, is gaining popularity among researchers (Noman et al., 2018a, Noman et al., 2018b; Nawaz et al., 2020; Ali et al., 2020a; Habib et al., 2021; Seleiman et al., 2021).

A review of previous literature reveals that the use of such chemicals for seed priming has the potential to accelerate the seed germination process and induce stress tolerance at later growth stages of plants (Johnson and Puthur, 2021). In this regard, different sugars (Mostofa et al., 2015; Shao et al., 2022), vitamins, antioxidants (Alam et al., 2022), plant-based extracts (Noman et al., 2018b; Ali et al., 2020d), and amino acids (Ahmed et al., 2019; Doycheva, 2022) are being applied individually or in combination. Several researchers have recommended the use of organic compounds for seed priming to obtain better production by stress amelioration (Ali et al., 2017; Habib et al., 2020; Saeed et al., 2023a). However, the potential of phytochemicals in modulating the cellular metabolism for stress tolerance is still unexplored.

Several secondary metabolites are synthesized by the plants, including alkaloids, flavonoids, phenols, and terpenoids, which have major contributions to the adaptations of plants to their surroundings (Montes et al., 2014). Alkaloids are specialized metabolites that influence numerous biological activities. Plants are protected by these naturally occurring nitrogenous chemicals in order to withstand a variety of stressful environmental situations (Bhambhani et al., 2021). Purine alkaloids, well-known secondary metabolites, are often used as mild stimulants. A well-known alkaloid 1,3,7-trimethylxanthine, commonly known as caffeine, originated from purine, belongs to the xanthene family, and is naturally present in almost 100 plant species (Montes et al., 2014). Caffeine is bitter, white, and crystalline in nature. It is found in coffee, tea, chocolates, and many other food items (Jadhav et al., 2016). It is similar in structure to cytokinins, which perform various metabolic functions in plants, especially the process of cell division (Taiz et al., 2015). Caffeine is reported as both enhancer and inhibitor of growth in plants (Montes et al., 2014). However, very little information is available on the metabolic role of caffeine, as a seed-treatment agent, in the process of seed germination and seedling emergence under drought stress. Recently, Emanuil et al. (2022) found the stress-relieving effect of exogenously applied caffeine in spinach plants. Moreover, the exogenous application of caffeine improved the yield of Bambara ground nut (*Vigna subterranea* L.), which was associated with caffeine-inducible better seed germination (Mshelmbula et al., 2018). Treatment of sunflower seeds with caffeine at low concentrations improved seed germination and seedling growth (Khursheed et al., 2009).

Among agronomic crops, wheat is the most widely traded product. It is the largest contributor with almost 50% and 30% in the world grain trade and grain production, respectively (Akter and Rafiqul Islam, 2017). Among cereals, it remains a major source of human diet for many civilizations in many parts of the world. Among cereals, wheat has secured the third position following rice and maize (Asseng et al., 2011). Approximately more than 35% of the global population, including Pakistan, depends on wheat to fulfill their food requirements. In Pakistan, almost one-third of the total cultivated land is rain-fed, receiving insufficient rainfall (Mahmood et al., 2021). In these regions, water deficiency is a major barrier to better production of wheat (Qayyum et al., 2011), and annually, approximately 30% loss in wheat yield occurs due to poor crop stand establishment.

In view of the information available, regarding the limited use of caffeine, it was hypothesized that exogenous use of caffeine as a seed priming agent may be helpful in ameliorating the negative impacts of PEG-induced drought stress on seed germination and seedling growth of wheat. The present study was designed with the objectives of exploring the possible roles of low doses of caffeine in improving seed germination and seedling emergence and vigor, in relation to activities of hydrolyzing enzymes, which have key roles in seed germination and seedling biomass production.

## 2 Materials and methods

### 2.1 Experimental conditions

The whole experiment was performed in a growth room at 25°C ± 2°C in the Experimental Botany Lab of the Department of Botany, Government College University Faisalabad. Light was maintained by a LED (PHILIPS cool daylight) light system. The given light intensity was 500 µmol m^−2^ s^−1^, which was maintained for 9 h on a daily basis. The experiment was arranged in a completely randomized design (CRD) with four replicates of each treatment. Wheat genotype SB1 (developed from a cross of S-24 and Bhakkar), with high yield potential, was used for the experimentation. The experiment comprised 48 Petri dishes, where 24 Petri dishes were allocated to non-stressed treatments, while the remaining 24 were allocated to drought-stressed ones. In each Petri dish, 30 seeds were sown using Whatman No. 1 filter paper in a double layer as a substrate. Hoagland’s nutrient solution (10 mL in each Petri dish) was supplied to the set of Petri dishes allocated to non-stressed treatments, and 18% PEG-8000 solution (10 mL in each Petri dish) prepared in Hoagland’s nutrient solution (with a solute potential of −0.65 MPa) was added in Petri dishes allocated to drought stress treatments. Hoagland’s nutrient solution was applied to make it compatible with the soil environment. PEG-induced drought stress reduced the availability of water to plants in a growth medium, and the plants showed similar physiological responses as observed under water-deficient conditions in soil (Gergely et al., 1980; Gonzalez et al., 2023). Seed priming treatments were as follows: no soaking (NS), water soaking (WS), and caffeine levels of 4 ppm, 8 ppm, 12 ppm, and 16 ppm. Before sowing, seeds were soaked for 12 h in water and each of the respective caffeine solutions, followed by air-drying to a constant weight. For surface sterilization, the seeds were treated with HgCl_2_ solution (0.1%) for 5 min and thoroughly washed with distilled water. After air-drying, 30 seeds were sown in each Petri dish supplied with respective solutions. The whole experiment was performed in duplicate. One set of the experiment was specified to measure the seed germination, seedling emergence, growth, and different physio-biochemical attributes, while the second set was specified to measure the activities of germination-related enzymes. After germination count in the first set of experiments, the seedlings were grown under the same environmental conditions for 15 days in the same Petri dishes to measure different biochemical attributes. After seed germination, the seedlings in the Petri dishes were supplied with the solutions of required strength at the rate of 20 mL per day in each Petri dish.

### 2.2 Seed germination-related attributes

Data on seed germination and related traits were recorded following the instructions of the Association of Official Seed Analysts (1990). Seed germination count was conducted on a daily basis and continued till the constant number was achieved. The seed germination percentage was calculated using the following equation.


$$\begin{array}{c} \text{Seed germination percentage}\\ \;=\left(\text{total seed germinated at final count}/\text{total seeds sown}\right)\times 100. \end{array}$$

Time to 50% seed germination (E_50_) was determined according to the method given by Coolbear et al. (1984), using the following equation.


$$\text{Time to }50\%\text{ seed germination}=t_{i}+\left[\left(N/2-n_{i}\right)\;\left(t_{j}-t_{i}\right)/n_{j}-n_{i}\right],$$

where *n_i_* and *n_j_* are the counts of seeds at the times *t_i_* and *t_j_*, respectively, that emerged from the adjacent counts, when *n_i_< N*/2 and *n_j_> N*/2. Here, *N* shows the total seeds that were germinated in the final count.

Mean emergence time (MET) was calculated using the following equation given by Ellis and Roberts (1981).


Mean emergence time=(ΣDn/Σ n).

Here, *n* corresponds to the emerged seeds on day *D*, and *D* corresponds to the number of days counted from the initiation of seed germination.

For the calculation of the coefficient of uniformity of emergence (CUE), the equation given by Bewley and Black (1985) was applied, as mentioned below.


$$\text{CUE}=\Sigma \;n/\Sigma \;\left[{\left(t^{/}-t\right)}^{2}\times n\right],$$

where *n* represents the number of germinated seeds with healthy emergence, counted on *t* day; *t* is the number of days after seed sowing; *t* ^/^ is the MET.

Seed emergence index (EI) was estimated following the method, given below, as described by the Association of Official Seed Analysts (1983).


$$\begin{array}{c} \text{EI}=\text{Count of germinated seeds}\\ \;+\text{days to first count}\ldots \ldots \ldots \text{ count of germinated seeds}\\ \;+\text{days to final count}. \end{array}$$

Germination energy (GE) is the percentage of seeds germinated at day 4 to the total number of seeds. Germination energy was calculated by the method devised by Ruan et al. (2002).

### 2.3 Activities of seed germination enzymes

For the estimation of the activities of enzymes, necessary for seed germination, the seeds grown in the second set of the experiment were used after 48 h of sowing.

To estimate the Amy activity, 10 germinating seeds per replicate were taken, ground well using a mortar and pestle in an ice-chilled solution of NaCl (1%), and prepared in phosphate buffer (0.2 mM) having pH 5.5 for the extraction of enzymes. The obtained homogenate was centrifuged for 20 min at 10,000 rpm. The supernatant obtained after centrifugation was used to measure the enzyme activity as reported by Chrispeels and Varner (1967). The Amy activity was measured as mg of starch hydrolyzed g^−1^ fresh weight h^−1^.

For the estimation of Prot activity in germinating seeds, the procedure given by Ainouz et al. (1972) was followed. From each replicate, five seedlings were selected, ground well using a mortar and pestle in an ice-cold solution of 1% NaCI, and prepared in 0.2 mM phosphate buffer having pH 7.0 for the extraction of enzymes. The finely ground homogenate was centrifuged for 20 min at 14,000 rpm. The supernatant (1 mL) was mixed well with 1% solution of casein (5 mL) and prepared using the phosphate buffer (0.2 M) with pH 6.0. The resultant solution was incubated for 1 h at 50°C. The reaction was terminated by adding 1 mL trichloroacetic acid (TCA) (40% solution) and mixing with Folin’s phenol reagent, and the absorbance was read using a spectrophotometer at 570 nm, following Classics Lowry et al. (1951).

For measuring the activity of Gluco in germinating wheat seeds, the mixture was prepared for incubation, containing maltose (0.1 mL) with the required strength, prepared using the McIlvaine buffer (Dawson et al., 1969) having pH 5. The same buffer (0.3 mL) was mixed with Gluco (0.1 mL) to prepare the Gluco solution. The extracted enzyme was mixed with this solution to start the reaction, followed by incubation at 37°C for 30 min. The Gluco activity was determined following Lloyd and Whelan (1969) using the glucose oxidase method, with the glucose liberated from maltose. Again, incubation was carried out at 37°C for 50 min, and 5 N HCl (2.5 mL) was added by continuous mixing to terminate the reaction. Then, the final prepared mixture was used to read the absorbance at 525 nm spectrophotometrically.

### 2.4 Caffeine content

Fresh root and shoot samples, stored at −20°C, were thawed using double-distilled water with a known quantity, and the supernatant was centrifuged at 10,000 *×g* at 4°C. The supernatant was then concentrated using gaseous nitrogen, followed by filtration using a 0.2-µm membrane Syringe Filter (Sartorius, Göttingen, Germany). The final obtained filtrate was then processed using HPLC (PerkinElmer, Waltham, MA, USA; Chromera 200 series USA), fitted with C18 column (Pinnacle DB Aqueous C18, 5 µm, 250 × 4.6 mm; Restek Corporation, Centre County, PA, USA). The separation was conducted using a 15% methanolic solution with a flow rate of 1 mL. The system was equipped with a Flexar UV/Vis LC detector. The quantitative analysis was conducted based on the obtained peak area against a series of known standards of 5–25 mg/kg.

### 2.5 Growth and morphological attributes

Data on the morphological parameters of plants were recorded after 15 days of the final germination count. Four plants were selected from each treatment and separated into roots and shoots. After washing, the excess water was removed from root surface. Fresh biomass and length of roots and shoots were noted, and the samples were oven-dried for 72 h at 65°C to measure the dry weights.

### 2.6 Biochemical parameters

#### 2.6.1 Photosynthetic pigments

The acetone extraction method was employed for the quantification of leaf chlorophyll and carotenoid (Car) contents. The leaf sample (0.1 g) was ground using a pestle and mortar in acetone (80%). The resulting material was centrifuged at 10,000 ×*g*, and the supernatant was used to read the absorbance at 663, 645, and 480 nm spectrophotometrically. Calculations for total chlorophyll (T. Chl.), chlorophyll *b* (Chl. *b*), and chlorophyll *a* (Chl. *a*) were made as described by Arnon (1949). Carotenoid content was measured using the method given by Kirk and Allen (1965).


$$\begin{array}{c} \text{T}.\text{ Chl}.({\text{mg g}}^{-1}\text{FW})\\ \;=[(0.0202\times \text{A}645)+(0.00802\times \text{A}663)]\times \text{V}/1,000\times \text{W}, \end{array}$$

$$\text{Chl}.\;b\;({\text{mg g}}^{-1}\text{FW})=\left[22.9\left(\text{A}645\right)-4.68\left(A663\right)\right]\times \text{V}/1,000\times \text{W},$$

$$\text{Chl}.\;a\;({\text{mg g}}^{-1}\text{FW})=\left[12.7\left(\text{A}663\right)-2.69\left(\text{A}645\right)\right]\times \text{V}/1,000\times \text{W},$$

$$\text{Car}.\;({\text{mg g}}^{-1}\text{FW})=\text{A}480+(0.114\times \text{A}663–0.638\times \text{A}645).$$

#### 2.6.2 Malondialdehyde and hydrogen peroxide

Malondialdehyde content in shoots and roots was estimated following the method given by Cakmak and Horst (1991). Leaf and root samples (0.50 g) of the same plant were ground separately using a pestle and mortar in 6% TCA (10 mL) by adding liquid nitrogen. After that, centrifugation was conducted at 10,000 ×*g* for 10 min, and the obtained supernatant (0.5 mL) was mixed with 5% TBA. The resultant mixture was heated at 95°C in a water bath. The reading of the final obtained material was taken at 600 nm and 532 nm spectrophotometrically. For H_2_O_2_ in roots and shoots, the above-obtained supernatant (0.5 mL each) was added in premixed 50 mM potassium phosphate buffer having pH 7.5 and 1 M KI, followed by incubation for 50 min. After that, the absorbance of the material was read at 390 nm, following the instructions given by Velikova et al. (2000).

### 2.7 Enzymatic antioxidants, total soluble proteins, and free amino acids

Fresh samples of roots and shoots (0.5 g) were ground with liquid nitrogen in 10 mL of ice-chilled 50 mM potassium phosphate buffer (pH 7.8). After centrifugation for 20 min at 10,000 ×*g* at 4°C, the supernatant was used to measure the antioxidative enzyme activities, free amino acids (FAA), and total soluble proteins (TSP).

The activity of superoxide dismutase (SOD) in shoots and roots of wheat seedlings was estimated using the method given by Giannopolitis and Ries (1977). Reaction solution (1 mL), containing enzyme extract, riboflavin (1.3 µM), methionine (13 mM), EDTA (75 nM), and NBT (50 µM), was prepared in formamide. The mixture was treated with light using a 20-W bulb for 15 min. A sample without any extract added was also prepared (blank sample) each time. The absorbance of the mixture was read using a spectrophotometer at 560 nm.

Activities of peroxidase (POD) and catalase (CAT) in shoots and roots were determined by following Chance and Maehly (1955). The reaction mixture (3 mL) for estimation of CAT activity was prepared using the same buffer as was used for the extraction of the enzymes. The absorbance of the mixture was measured in a time-scale manner. The absorbance of the mixture was read at 240 nm with intervals of 20 s up to 120 s. For the activity of POD, the reaction mixture was prepared by mixing 40 mM H_2_O_2_, 100 µL enzyme extract, and 50 mM buffer as was used for the extraction of enzymes and 20 mM guaiacol. The absorbance of the reaction mixture was read at 470 nm in a time scan manner for 2 min with 20-s intervals. The activity of ascorbate peroxidase (APX) was determined using the method of Asada and Takahashi (1987). The reaction mixture contained 300 µL ascorbate (0.5 mM), 300 µL H_2_O_2_, phosphate buffer (2.1 mL), and 300 µL enzyme extract. The absorbance of the final prepared mixture was measured spectrophotometrically at 290 nm in a time scan manner with intervals of 20 s for 2 min.

For the estimation of glutathione reductase (GR) activity in roots and shoots, the procedure described by Schaedle and Bassham (1977) was followed. A sample of 0.2 g was ground in 5 mL of 50 mM Tris-HCl, followed by centrifugation at 22,000 ×*g* for 4 min. The reaction mixture was prepared by adding 200 µL supernatant, 50 mM Tris buffer (pH 7.6), glutathione disulfide (1 mM), NADPH (0.15 mM), and MgCl_2_ (mM). A decrease in absorbance of NADPH was recorded at 340 nm using a spectrophotometer. The GR activity was measured in units per milligram of proteins.

### 2.8 Non-enzymatic antioxidants

Total phenolic content (TPC) was determined by using the Folin–Ciocâlteu phenol reagent, as described by Julkunen-Titto (1985). The fresh samples (0.05 g) were well homogenized in acetone (80% prepared with dH_2_O) and centrifuged for 10 min at 10,000 ×*g*. The supernatant (100 µL) was mixed thoroughly with 1 mL of Folin–Ciocâlteu phenol reagent, and 2 mL of ddH_2_O was added to the mixture. After that, 20% Na_2_CO_3_ (5 mL) was added to the triturate. Finally, the volume of the mixture was made to 10 mL by adding ddH_2_O, and the absorbance was read at 750 nm. For the quantification of leaf TPC in the samples, an absorbance curve was made using standard solutions of tannin with known concentration. The total flavonoid content (TFC), in shoots and roots of seedlings, was assayed following the method given by Sultana et al. (2009). To the methanolic extract (1 mL), 5% NaNO_2_ (0.3 mL) and 1 M NaOH (2 mL) were added. After 10 min, 2.8 mL ddH_2_O was added to the mixture and incubated for 40 min at room temperature. The absorbance was read spectrophotometrically at 430 nm of the final prepared triturate. For quantifying TFC, a series of known standards of catechin were used.

The total anthocyanin (TAC) content in roots and shoots of wheat seedlings was determined using the methanolic extraction method given by Mirecki and Teramura (1984). Shortly, 0.5 g of fresh material of root and leaf was ground separately in 10 mL of acidic methanol. After centrifugation for 5 min at 14,000 ×*g*, the absorbance of the supernatant was measured spectrophotometrically at 657 nm and 530 nm. Total anthocyanin content was quantified using the following equation.


$$\text{TAC}=\text{A}657\times \left(\text{A}530-0.25\right)\times {\text{M}}^{-1}.$$

Here, M represents the fresh root and shoot biomass (Thiobarbituric acid) in grams used for the extraction, and A530 and A657 are the absorptions at the specific wavelengths.

Determination of ascorbic acid (AsA) in roots and shoots of the seedlings was carried out following the protocol described by Mukherjee and Choudhuri (1983). The sample (0.5 g) was ground using a pestle and mortar in 6% TCA solution and centrifuged for 20 min at 10,000 ×*g*. The supernatant (4 mL) was mixed with 2% DNPH (2 mL) and prepared in the acidic medium. A drop of thiourea (10%) was added to the resultant mixture and then incubated at 95°C with the addition of 80% H_2_SO_4_ (v/v). The final prepared solution was read at 530 nm spectrophotometrically.

For the estimation of reduced glutathione (GSH) and oxidized glutathione (GSSG) in roots and shoots of wheat seedlings, the method given by Griffith (1980) was used. Shortly, fresh material of root or shoot (250 mg) was well homogenized using 0.1 M HCl (2 mL) and EDTA (1 mM) in a pestle and mortar. After that, the supernatant was obtained by centrifugation for 15 min at 12,000 ×*g* at 4°C. For preparing the reaction mixture, the following were added: 200 µL of the phosphate buffer (strength of 125 µM), having EDTA of 6.3 mM with pH 7.5, 100 µL DTNB of 6.0 mM strength, 200 µL extract, and 500 µL NADPH (0.3 mM). The absorbance of the final prepared mixture was read spectrophotometrically at 412 nm.

### 2.9 Osmolytes

Free proline (Pro) content, in roots and shoots, was assayed following the method of Bates et al. (1973). Fresh biomass of roots and shoots (0.5 g) was ground separately by adding 10 mL of 3% sulfosalicylic acid using a pestle and mortar. After centrifugation of the homogenate, the supernatant (2.0 mL) was mixed with acid ninhydrin (2 mL). Acidic ninhydrin was prepared using 1.25 g ninhydrin in 30 mL glacial acetic acid, 6 M orthophosphoric acid, and 2 mL of glacial acetic acid in a test tube. The final prepared mixture was then incubated for 1 h at 100°C using a water bath. Toluene (4 mL) was added to the cooled mixture and passed through the air stream for 1–2 min. The toluene layer of the mixture was taken, and the absorbance was read spectrophotometrically at 520 nm. Quantification of the Pro was made by forming an absorbance curve with pure standards of proline using the given formula:


$$\begin{array}{c} {\text{µmol Pro g}}^{-1}\;fresh\;weight\\ \;=\left({\text{µg proline mL}}^{-1}\times \text{mL of toulene}/11.5\right)/\left(\text{g of sample}\right). \end{array}$$

Glycine betaine (GB), in shoots and roots of seedlings, was assessed following Grieve and Grattan (1983). Shortly after, dry material of root and shoot samples (5 g) was ground separately using ddH_2_O (10 mL). After centrifugation of the homogenate for 10 min at 5,000 ×*g*, 1 mL of 2 N HCL was mixed with 1 mL of supernatant. Potassium triiodide solution (200 µL) was mixed with 0.5 mL of the prepared mixture and put in chilled water with shaking. Later on, 2 mL ddH_2_O (ice cooled) and 20 mL 1,2-dichloromethane were added to the cooled mixture and put in chilled water. After that, the mixture was treated with continuous air stream for 1–2 min in an ice bath. The absorbance of the lower layer was read spectrophotometrically at 365 nm. GB, in roots and shoots of seedlings, was quantified based on the standard curve obtained by using pure GB standard solutions (5–25 ppm) of analytical grade.

### 2.10 Metabolites

TSP content was determined using the same phosphate buffer extract, as used for the analysis of enzymatic antioxidants (Bradford, 1976). Shortly, 2 mL of Bradford’s reagent was added to 0.1 mL of the enzyme extract and mixed well, and the absorbance was read spectrophotometrically at 595 nm. Quantification of TSP was conducted by following a standard curve made from pure bovine serum albumin (200–14,000 mg/kg).

Determination of FAA in roots and shoots was conducted using the same extract as was used for enzymatic antioxidants and TSP, following the methodology of Hamilton and Van Slyke (1943). Briefly, 10% of the pyridine solution was mixed with 1 mL of buffer-extracted supernatant and ninhydrin (2%) solution. Then, the mixture obtained was incubated for approximately 30 min at room temperature. After that, the absorbance of the final prepared mixture was read at 570 nm spectrophotometrically. The content of FAA in roots and shoots was quantified using the standard curve.

Determination of total soluble sugars (TSS) in roots and shoots was conducted using the procedure described by DuBois et al. (1956). Fresh material (0.5 g) of roots and shoots was extracted separately using methanol. After centrifugation, 0.1 mL of the supernatant was reacted with 3 mL of anthrone reagent. The mixture was then heated for 15 min, using a water bath, at 95°C. After cooling the mixture in ice-chilled water, it was incubated for 30 min at room temperature. The absorbance was then read at 490 nm. The quantification of the TSS contents in roots and shoots was computed using a standard curve prepared with a range of standard solutions (200–1,000) of pure sugar of analytical grade.

Total reducing sugars (TRS) were determined in shoots and roots using the method of Wood and Bhat (1988). The sample (0.5 g) was extracted using 80% methanolic solution by grinding. The material was then centrifuged at 14,000 ×*g* for 15 min at room temperature, and the supernatant was obtained. To 1 mL of the supernatant, DNS (4 mL) was added, and the material was heated for 5 min using a water bath at 95°C. The mixture was then cooled using ice-chilled water. The mixture was incubated at room temperature, and the absorbance was measured at 540 nm spectrophotometrically. A formula given by Lakho et al. (2017) was adopted to uncover the NRS content.


TSS=Non-reducing sugars+reducing sugars.

### 2.11 Statistical analysis

The experiment was carried out using a CRD. The CoStat Computer Program (Windows version 6.303, PMB 320, Monterey, CA, USA) was employed to estimate significant variations in the studied parameters among treatments. For the estimation of significant variations among the means of studied parameters, the least significant difference (LSD) test with a 5% significance level was employed. Furthermore, in order to access correlations among the studied attributes, the principal component analysis (PCA) was employed, and a heatmap was drawn to detect the correlations among studied parameters using the RStudio version 4.2.2 computer program.

## 3 Results

**Figure 1:**
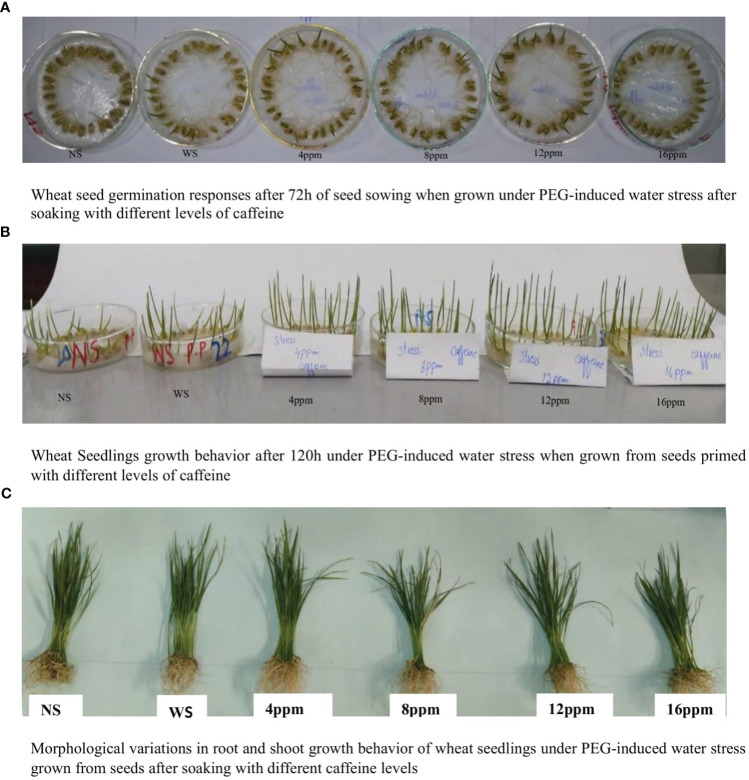
Seed germination and seedling growth behavior of wheat under PEG-induced water stress when grown after soaking in different levels of caffeine. **(A)** Wheat seed germination responses after 72 h of seed sowing when grown under PEG-induced water stress after soaking with different levels of caffeine. **(B)** Wheat seedling growth behavior after 120 h under PEG-induced water stress when grown from seeds primed with different levels of caffeine. **(C)** Morphological variations in root and shoot growth behavior of wheat seedlings under PEG-induced water stress grown from seeds after soaking with different caffeine levels.

**Figure 2:**
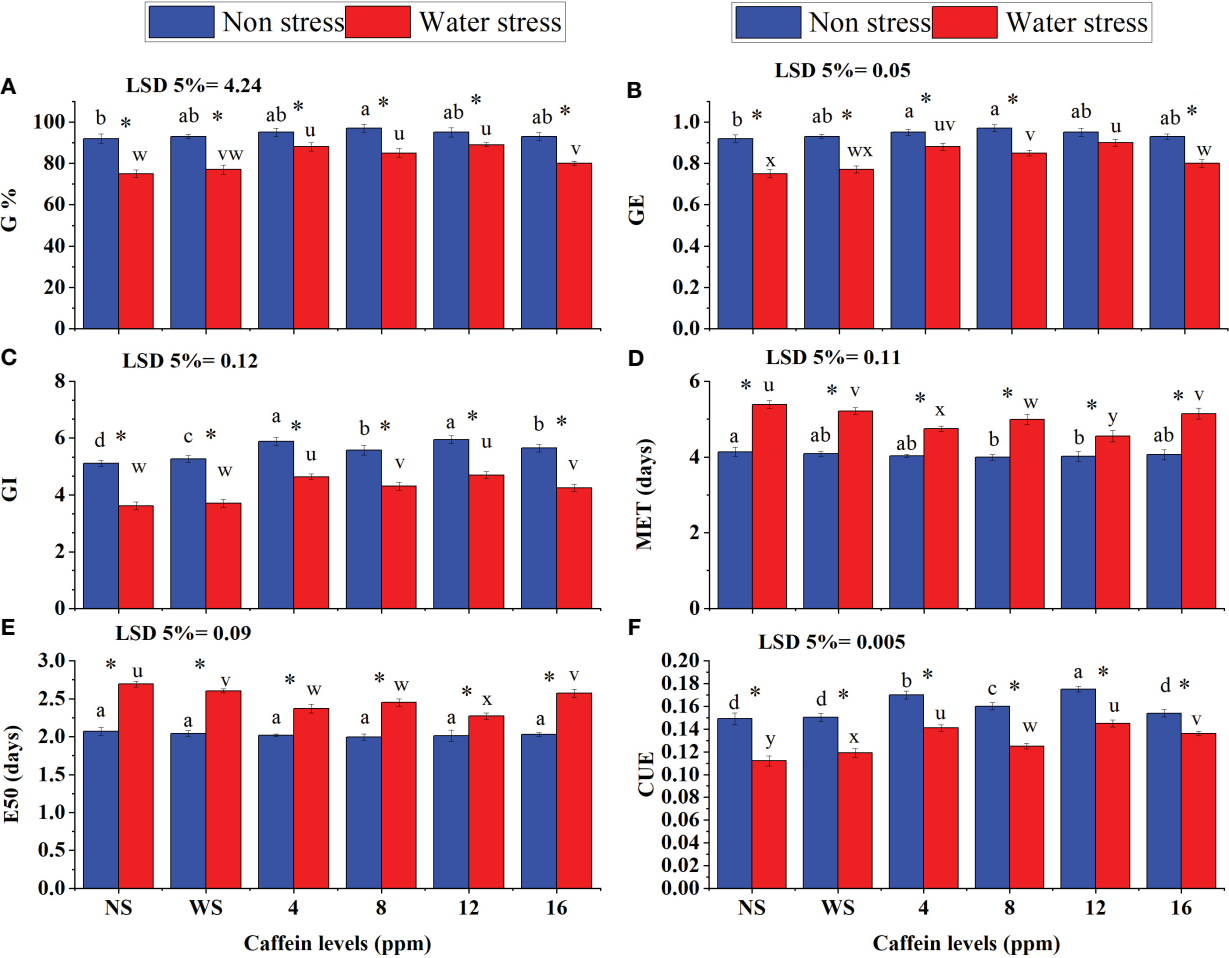
Seed G% **(A)**, GE **(B)**, GI **(C)**, MET, **(D)**, E_50_ **(E)**, and CUE **(F)** of wheat grown from seeds treated with different levels of caffeine under non-stress and PEG-induced water stress (mean ± SE; *n* = 4). Bars with the same alphabets do not differ significantly [blue for non-stressed ones (a, b, c, etc.) and red for stressed ones (u, v, w, etc.) against a specific caffeine treatment]. * on bars showing the significant difference between stress (red) and non-stress (blue) in a specific treatment. NS, no soaking; WS, water soaking; G%, germination percentage; E_50_, time to 50% seed germination; MET, mean emergence time; CUE, coefficient of uniformity of emergence; GI, germination index; GE, germination energy. P values for G%, GE, GI, MET, E50 and CUE are P ≤ 0.001**, P ≤ 0.0019**, P ≤ 0.0007***, P ≤ 0.0004***, P ≤ 0.0004*** and P ≤ 0.0086** respectively.

The impacts of PEG-induced water stress on seed germination attributes and seedling vigor are presented in **Figures 1**, **2**. The visual ameliorative impacts of caffeine seed treatments on seed germination and seedling vigor are presented in **Figures 1A, B**. Drought stress, imposed by PEG-8000, adversely affected the GI, GE, MET, E_50_, G%, and CUE (**Figures 2A–F**). Pre-sowing seed treatment with caffeine showed significant improvements in all germination-related attributes of wheat seeds when grown under drought stress and normal moisture conditions. However, the extent of amelioration was caffeine dose-specific. Caffeine doses of 4 ppm and 12 ppm were the most effective ones in mitigating the adverse effects of moisture deficit on G%, GE, GI, and CUE. However, the higher level of caffeine, i.e., 16 ppm, was the least effective in the case of G%, GE, GI, and CUE, whereas 12-ppm caffeine level was the most effective in the case of MET and E_50_. Caffeine seed priming also improved the germination-related attributes of seeds under normal conditions, but the impact was less pronounced than that of water-deficit conditions (**Figures 2A–F**).

**Figure 3:**
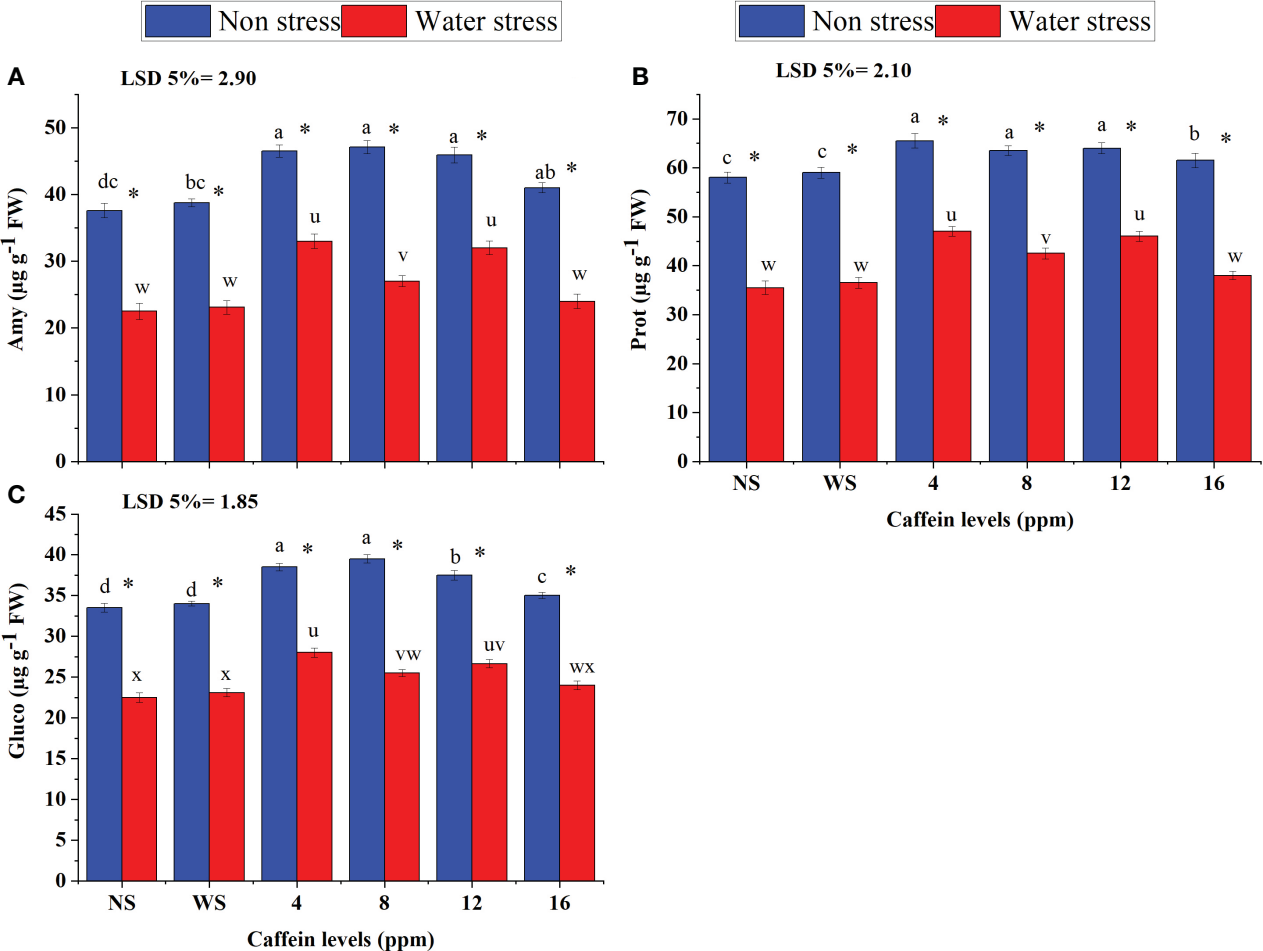
Amyl **(A)**, Prot **(B)**, and Gluco **(C)** of wheat grown from seeds treated with different levels of caffeine under non-stress and PEG-induced water stress (mean ± SE; *n* = 4). Bars with the same alphabets do not differ significantly [blue for non-stressed ones (a, b, c, etc.) and red for stressed ones (u, v, w, etc.) against a specific caffeine treatment]. * on bars showing the significant difference between stress (red) and non-stress (blue) in a specific treatment. NS, no soaking; WS, water soaking; Amy, amylase; Prot, protease; Gluco, glucosidase. P values for Amy, Prot, GIuco, are ≤ 0.0079**, P ≤ 0.0085** and P ≤ 0.0084** respectively.

Moisture deficiency also adversely affected the activities of enzymes related to germination, including Amy (**Figure 3A**), Prot (**Figure 3B**), and Gluco (**Figure 3C**). Seed priming with caffeine ameliorated the deleterious impacts of water scarcity on Amy, Prot, and Gluco in a dose-dependent manner. Caffeine seed priming also improved the activities of these enzymes under non-stress conditions. Under drought stress, seed treatment with caffeine levels of 4 ppm and 12 ppm, followed by 8 ppm, showed better results in improving the activities of Prot, Amy, and Gluco, than other doses. However, under non-stressed conditions, 4-ppm, 8-ppm, and 12-ppm caffeine levels were found equally effective in improving the activities of Amy and Prot, but for Gluco activity, more improvement was found due to 4- and 8-ppm doses followed by 12 ppm caffeine. However, a higher level of caffeine (16 ppm) was not found to be very effective under both drought stress and well-watered conditions (**Figures 3A–C**).

**Figure 4:**
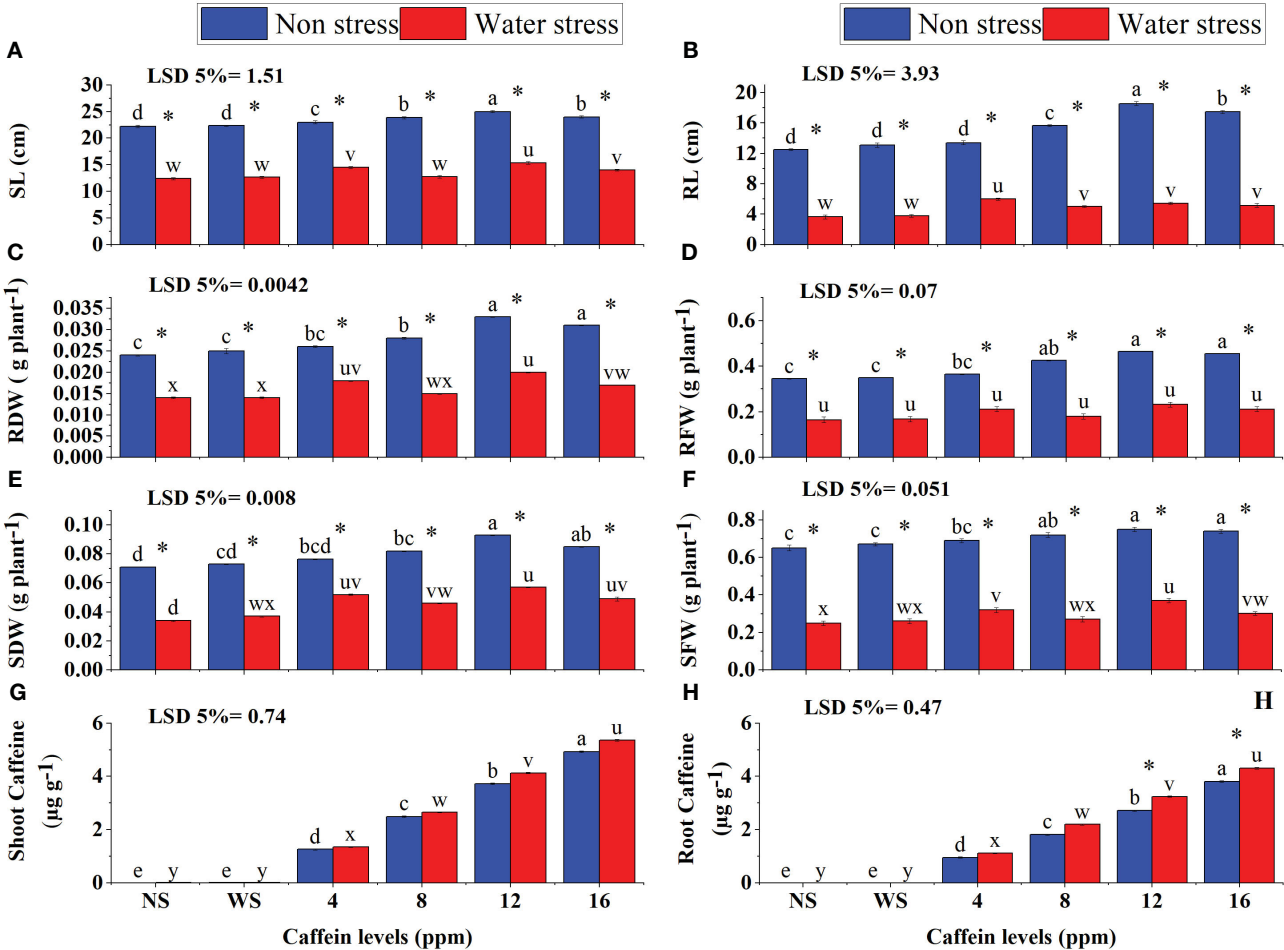
SL **(A)**, RL **(B)**, RDW **(C)**, RFW **(D)**, SDW **(E)**, SFW **(F)**, shoot caffeine **(G)**, and root caffeine **(H)** of wheat seedlings grown from seeds treated with different levels of caffeine under non-stress and PEG-induced water stress (mean ± SE; *n* = 4). Bars with same alphabets do not differ significantly [blue for non-stressed ones (a, b, c, etc.) and red for stressed ones (u, v, w, etc.) against a specific caffeine treatment]. * on bars showing the significant difference between stress (red) and non-stress (blue) in a specific treatment. NS, no soaking; WS, water soaking; SL, shoot length; RL, root length; RFW, root fresh weight; RDW, root dry weight; SFW, shoot fresh weight; SDW, shoot dry weight. P values for SL, RL, RDW, RFW, SDW, SFW, Shoot Caffeine and Root Caffeine are P ≤ 0.0031**, P ≤ 0.0001***, P ≤ 0.00351**, P ≤ 0.0001***, P ≤ 0.0088**, P ≤ 0.0024**, P ≤ 0.0002*** and P ≤ 0.00013*** respectively.

Data on growth parameters show that drought stress severely affected the root length (RL), shoot length (SL), root fresh weight (RFW), shoot fresh weight (SFW), root dry weight (RDW), and shoot dry weight (SDW) of wheat seedlings (**Figures 4A–F**). Seed priming with caffeine considerably improved the growth traits of seedlings under stress in a dose-specific manner. Pre-sowing seed treatment with 12 ppm, followed by 8 ppm and 16 ppm, of caffeine produced the most promising results in increasing biomass production. The maximum increase in SFW (13%) and SDW (24%) was induced by 12 ppm, followed by 16 ppm, of caffeine treatment under stress. However, the least increment was induced by 4 ppm caffeine. Caffeine treatment (4 ppm and 12 ppm) considerably increased SL under drought stress, followed by 16-ppm treatment (**Figure 4A**). Caffeine seed treatment also significantly improved the growth attributes of seedlings grown under well-watered conditions, but the impact was less pronounced as compared with the stressed plants.

Seed treatment with caffeine significantly increased the caffeine contents in roots and shoots of wheat seedlings under both water regimes. Drought-stressed seedlings showed higher caffeine content in shoots and roots than those grown under a normal moisture supply. Shoots show higher caffeine content than roots under both stress and normal conditions. The results revealed a high rate of translocation of caffeine from root to shoot after germination (**Figures 4G, H**).

**Figure 5:**
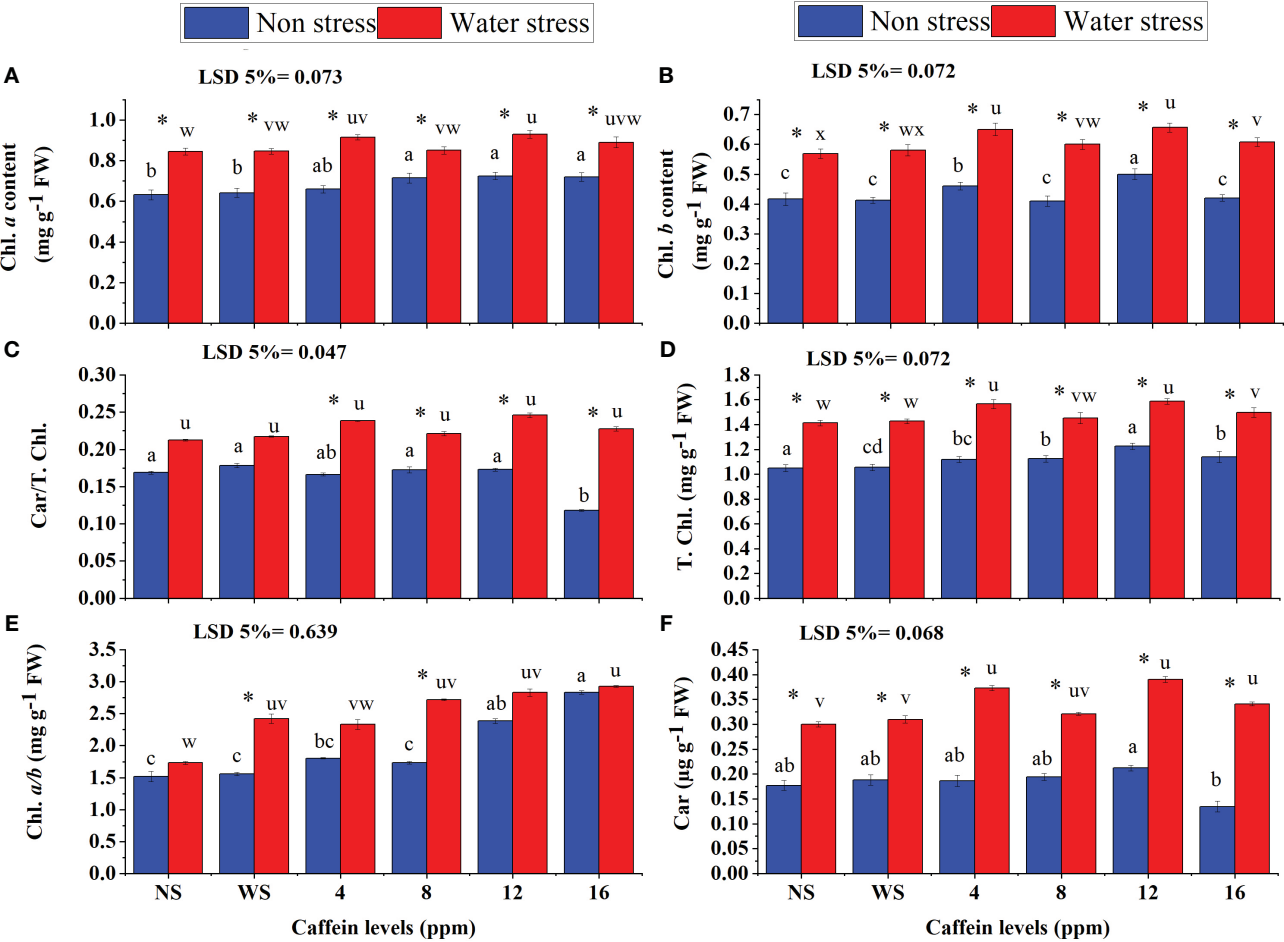
Leaf Chl. *a* **(A)**, Chl. *b* **(B)**, T. Chl. **(D)**, Chl. *a*/*b* **(E)**, Car. **(F)**, Car/T., and Chl. **(C)** of wheat seedlings grown from seeds treated with different levels of caffeine under non-stress and PEG-induced water stress (mean ± SE; *n* = 4). Bars with same alphabets do not differ significantly [blue for non-stressed ones (a, b, c, etc.) and red for stressed ones (u, v, w, etc.) against a specific caffeine treatment]. * on bars showing the significant difference between stress (red) and non-stress (blue) in a specific treatment. NS, no soaking; WS, water soaking; Chl. *a*, chlorophyll *a*; Chl. *b*, chlorophyll *b*; T. Chl., total chlorophyll; Chl. *a*/*b*, chlorophyll *a*/*b* ratio; Car, carotenoid; Car./T. Chl., carotenoid/total chlorophyll ratio; MDA, malondialdehyde. P values for Chl. a, Chl b, Car/T. Chl., T. Chl., Chl. a/b and Car are P ≤ 0.0001***, P ≤ 0.0042**,P ≤ 0.0019**,P ≤ 0.0000***, P ≤ 0.029* and P ≤ 0.0002*** respectively.

Caffeine seed treatment significantly increased the photosynthetic pigments (Chl. *a*, Chl. *b*, Car./T. Chl., Chl. *a*/*b*, Car., and T. Chl.) in wheat seedlings under both water deficit and normal conditions (**Figures 5A–F**). Regarding, Chl. *b* (**Figure 5B**), Car./T. Chl. (**Figure 5C**), Chl. *a* (**Figure 5A**), Car. (**Figure 5F**), and T. Chl. (**Figure 5D**), considerable increments were recorded in seedlings raised from seeds primed with 12 ppm caffeine, followed by 4 ppm. However, in the case of Chl. *a*/*b* (**Figure 5E**), 16 ppm caffeine showed the highest increase, followed by 12 ppm at both water regimes. Moreover, 16-ppm caffeine treatment significantly decreased Car./T. Chl. in plants grown in well-watered conditions.

**Figure 6:**
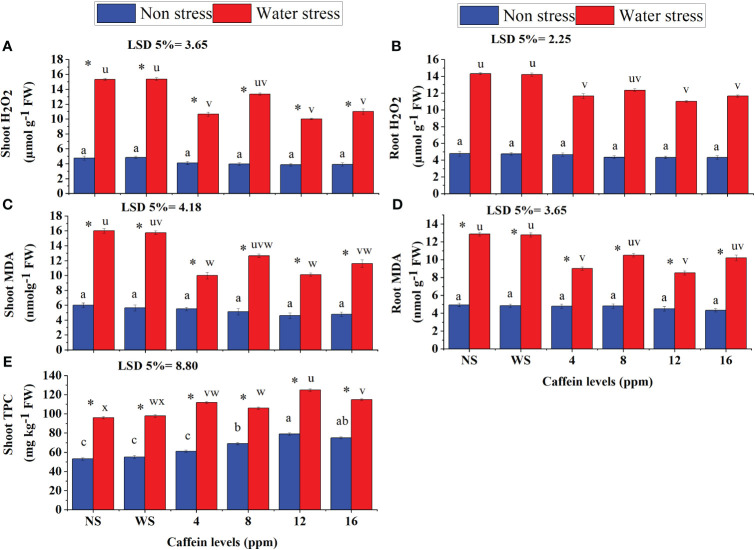
Shoot TPC content **(E)**, MDA **(A, B)**, and H_2_O_2_ **(C, D)** in shoot and root of wheat seedlings grown from seeds treated with different levels of caffeine under non-stress and PEG-induced water stress (mean ± SE; *n* = 4). Bars with same alphabets do not differ significantly [blue for non-stressed ones (a, b, c, etc.) and red for stressed ones (u, v, w, etc.) against a specific caffeine treatment]. * on bars showing the significant difference between stress (red) and non-stress (blue) in a specific treatment. NS, no soaking; WS, water soaking; MDA, malondialdehyde; H_2_O_2_, hydrogen peroxide; TPC, total phenolic content. P values for Shoot H202, Root H2O2, Shoot MDA, Root MDA, Shoot TPC are P ≤ 0.0002***, P ≤ 0.0000***,P ≤ 0.0005***, P ≤ 0.0004*** and P ≤ 0.0009*** respectively.

Caffeine seed treatment significantly reduced MDA and H_2_O_2_ accumulation in roots and shoots of seedlings grown in a water deficit environment (**Figures 6A–D**). Among different caffeine levels, 12-ppm level proved to be more effective followed by 4-ppm level. In contrast, in reducing root H_2_O_2_ content, 16-ppm level of caffeine proved better than 4- and 8-ppm levels (**Figure 6**).

Total phenolic contents in shoots of seedlings increased significantly when grown in water stress (**Figure 6E**). Caffeine treatment further increased the shoot TPC contents in wheat seedlings. This caffeine-induced increase in shoot TPC was caffeine level-specific. TPC in shoot increased at a maximum level by 12-ppm caffeine dose, followed by 8- or 16-ppm dose (**Figures 5**, **6**).

**Figure 7:**
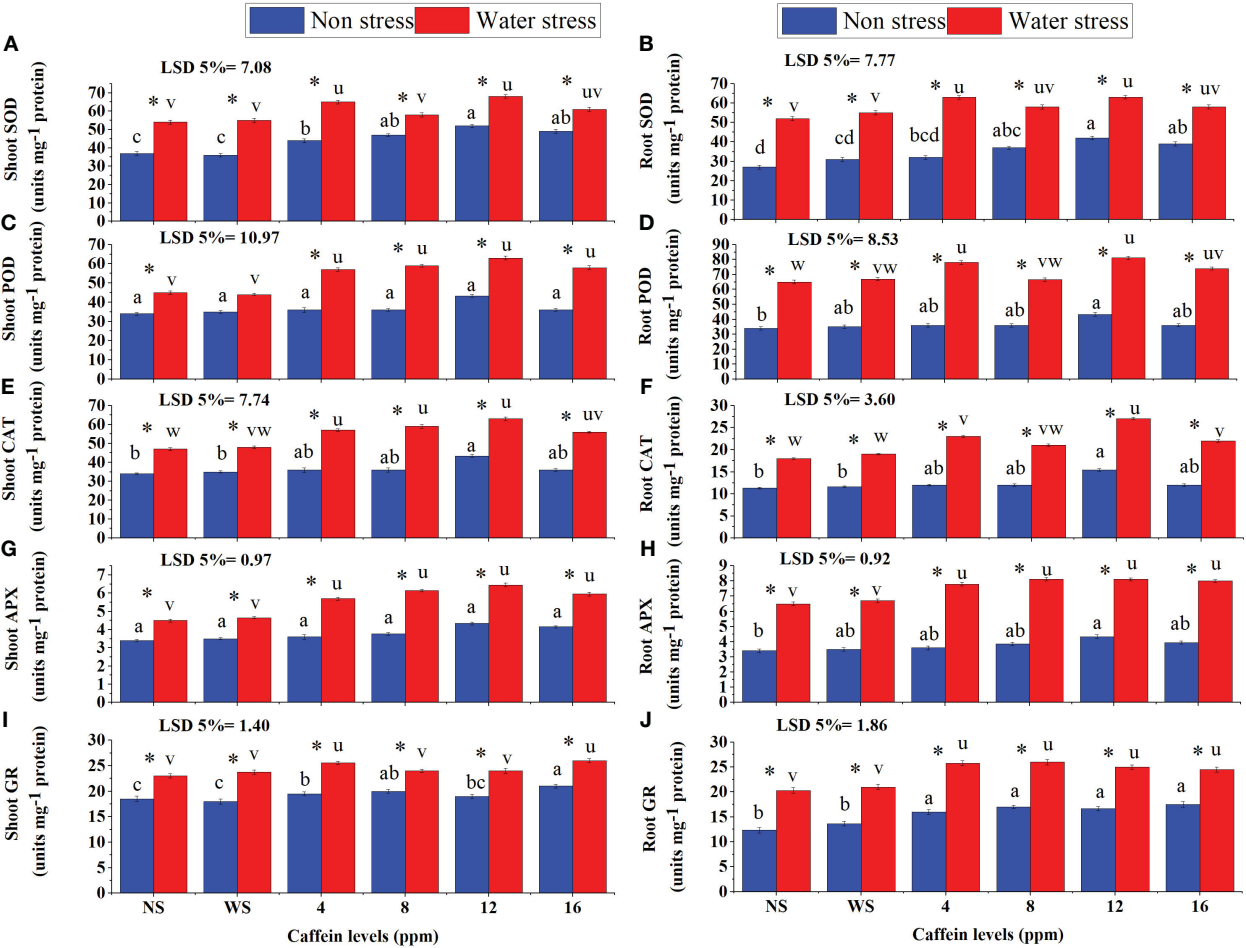
SOD **(A, B)**, POD **(C, D)**, CAT **(E, F)**, APX **(G, H)**, and GR **(I, J)** activities in shoot and root, respectively, of wheat seedlings grown from seeds treated with different levels of caffeine under non-stress and PEG-induced water stress (mean ± SE; *n* = 4). Bars with same alphabets do not differ significantly [blue for non-stressed ones (a, b, c, etc.) and red for stressed ones (u, v, w, etc.) against a specific caffeine treatment]. * on bars showing the significant difference between stress (red) and non-stress (blue) in a specific treatment. NS, no soaking; WS, water soaking; SOD, superoxide dismutase; POD, peroxidase; CAT, catalase; APX, ascorbate peroxidase; GR, glutathione reductase. P values for Shoot SOD, Root SOD, Shoot POD, Root POD, Shoot CAT, Root CAT, Shoot APX, Root APX, Shoot GR and Root GR are P ≤ 0.0016**, P ≤ 0.000***, P ≤ 0.0008***, P ≤ 0.0000***, P ≤ 0.0014**, P ≤ 0.0009***, P ≤ 0.0005***, P ≤ 0.0000***, P ≤ 0.013* and P ≤ 0.0015** respectively.

Drought stress significantly increased the activities of SOD, POD, CAT, APX, and GR in roots and shoots of seedlings (**Figures 7A–J**). Caffeine seed treatment further enhanced the activities of these enzymes in roots and shoots of seedlings under drought stress. Caffeine seed priming also increased the activities of these enzymes in normally grown plants. However, the extent of increment in enzyme activities in roots and shoots of wheat seedlings, grown under either drought stress or non-stress conditions, was caffeine level-specific. Seed treatment with 12 ppm caffeine increased the POD and SOD activities more than other doses, in both roots and shoots of seedlings under drought stress, followed by 4- and 8-ppm levels, except the shoot POD, where all caffeine doses showed the same increment in its activity. Seed treatment with 12 ppm caffeine under water deficit slightly increased the activity of CAT in shoots (**Figure 7E**). However, the same dose of caffeine (12 ppm) caused a significant enhancement in the activity of CAT in roots of seedlings under drought stress (**Figure 7F**). All caffeine doses showed similar effectiveness in improving APX activity in shoots and roots of seedlings, facing moisture scarcity (**Figures 7G, H**). Moreover, it was recorded that seed treatment with 12 ppm caffeine significantly increased the activity of enzymes in roots and shoots of well-watered wheat plants too, in comparison with the rest of the doses of caffeine.

The activity of GR considerably increased in roots and shoots of seedlings under moisture deficit conditions (**Figures 7I, J**). Pre-sowing seed treatment with caffeine further improved GR activity. Seed treatment with 4 ppm and 16 ppm caffeine induced a higher increase in shoot GR activity. For root GR activity, 4-ppm dose performed better but differed non-significantly with other levels of caffeine. Under non-stressed conditions, an increase in the activity of GR was more prominent in roots and shoots of seedlings grown from seeds primed with 16 ppm caffeine than in other doses.

**Figure 8:**
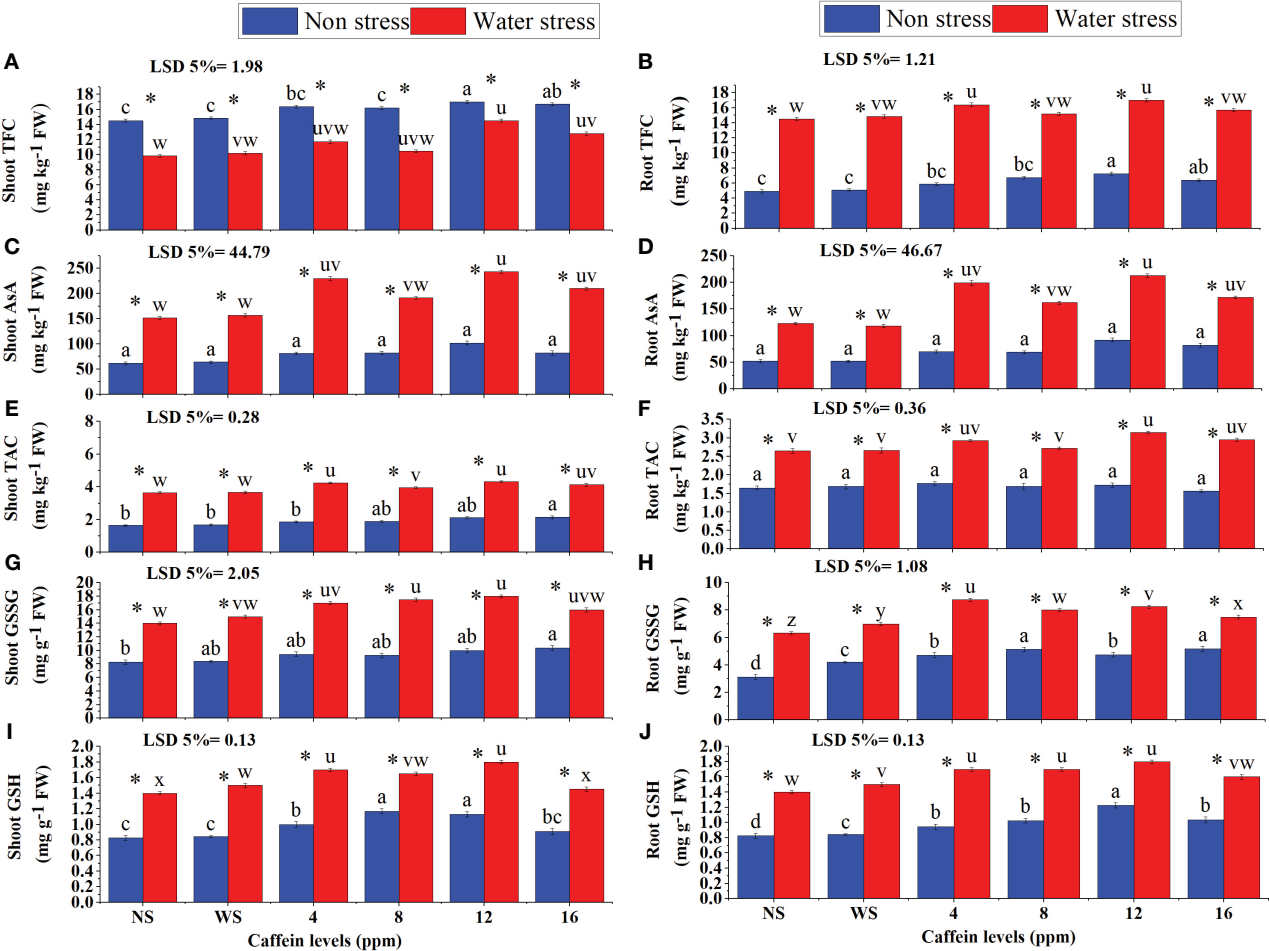
Flavonoids **(A, B)**, AsA **(C, D)**, anthocyanin **(E, F)**, GSSG **(G, H)**, and GSH **(I, J)** in shoot and root, respectively, of wheat seedlings grown from seeds treated with different levels of caffeine under non-stress and PEG-induced water stress (mean ± SE; *n* = 4). Bars with same alphabets do not differ significantly [blue for non-stressed ones (a, b, c, etc.) and red for stressed ones (u, v, w, etc.) against a specific caffeine treatment]. * on bars showing the significant difference between stress (red) and non-stress (blue) in a specific treatment. NS, no soaking; WS, water soaking; AsA, ascorbic acid; GSH, reduced glutathione; GSSG, oxidized glutathione. P values for Shoot TFC, Root TFC, Shoot AsA, Root AsA, Shoot TAC, Root TAC, Shoot GSSG, Root GSSG, Shoot GSH and Root GSH are P ≤ 0.033*, P ≤ 0.028*,P ≤ 0.0001***, P ≤ 0.0003***, P ≤ 0.0000*** , P ≤ 0.0000***, P ≤ 0.0000***, P ≤ 0.0290*, P ≤ 0.0091** and P ≤ 0.0057** respectively.

Data showed a remarkable increase in TFC, AsA, TAC, GSSG, and GSH contents in roots and shoots of drought-stressed plants (**Figure 8**). Seed treatment with different concentrations of caffeine further enhanced the contents of these non-enzymatic antioxidants. Caffeine seed treatment also improved the contents of these non-enzymatic antioxidants in roots and shoots of wheat seedlings grown in well-watered conditions. In water-stressed plants, 12-ppm caffeine treatment caused the highest increase in these antioxidants, followed by 4-ppm level, except for the root GSSG, where 4-ppm caffeine dose stood out from the rest of the treatments. Under non-stressed conditions, 12-ppm caffeine dose considerably increased TFC (**Figures 8A, B**) and AsA (12 ppm) in shoots and roots. However, shoot TAC was positively influenced by 16-ppm caffeine level. For root and shoot GSSG, 16-ppm level of caffeine performed better than other treatments. However, for shoot GSH (**Figure 8I**), 12 ppm and 8 ppm were taken as superior ones, and for root GSH (**Figure 8J**), 12-ppm caffeine level was taken as the superior one (**Figure 8**).

**Figure 9:**
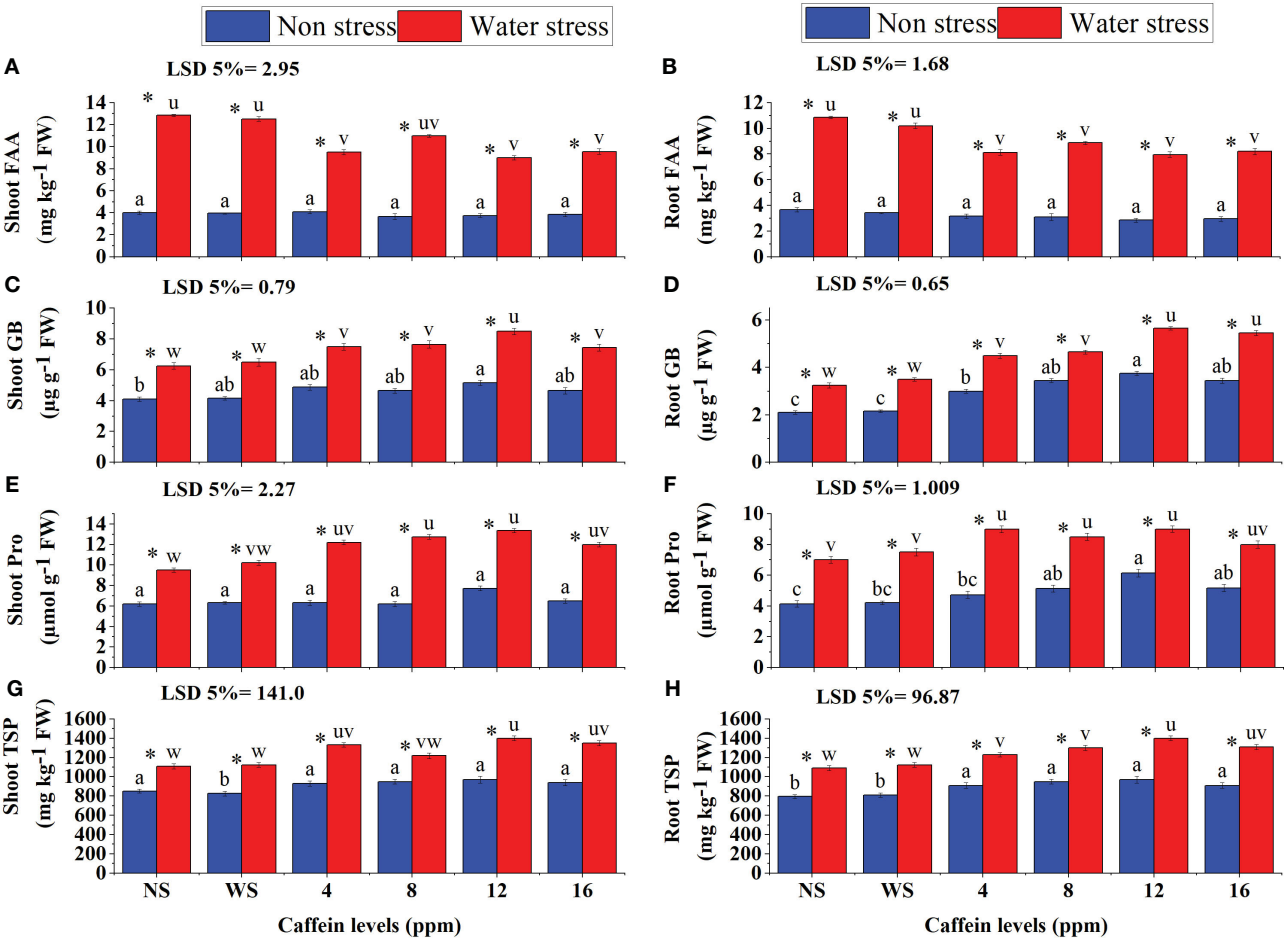
FAA **(A, B)**, GB **(C, D)**, Pro **(E, F)**, and TSP **(G, H)** in root and shoot, respectively, of wheat seedlings grown from seeds treated with different levels of caffeine under non-stress and PEG-induced water stress (mean ± SE; *n* = 4). Bars with same alphabets do not differ significantly [blue for non-stressed ones (a, b, c, etc.) and red for stressed ones (u, v, w, etc.) against a specific caffeine treatment]. * on bars showing the significant difference between stress (red) and non-stress (blue) in a specific treatment. NS, no soaking; WS, water soaking; FAA, free amino acids; GB, glycine betaine; Pro, proline; TSP, total soluble proteins; TPC, total phenolic content. P values for Shoot FAA, Root FAA, Shoot GB, Root GB, Shoot Pro, Root Pro, Shoot TSP and Root TSP are P ≤ 0.0001***, P ≤ 0.0000***, P ≤ 0.021*, P ≤ 0.0021**, P ≤ 0.0002***, P ≤ 0.0000***, P ≤ 0.0048** and P ≤ 0.0074** respectively

The contents of FAA, GB, Pro, and TSP in roots and shoots and TPC in shoots of seedlings increased significantly in drought stress (**Figure 9**). Caffeine treatment further increased GB, Pro, and TSP levels in both shoots and roots and TPC contents in shoot of wheat seedlings. However, caffeine seed priming decreased the FAA in roots as well as in shoots of wheat seedlings. This reduction in FAA and increase in GB, Pro, and TSP content in roots and shoots and TPC in shoot, due to seed priming, were caffeine level-specific. Seed treatment with 12 and 16 ppm caffeine decreased FAA (**Figures 9A, B**) in roots and shoots. GB, Pro, and TSP in roots and shoots and TPC in shoot increased maximally with 12-ppm caffeine dose, followed by 8 ppm or 16 ppm.

**Figure 10:**
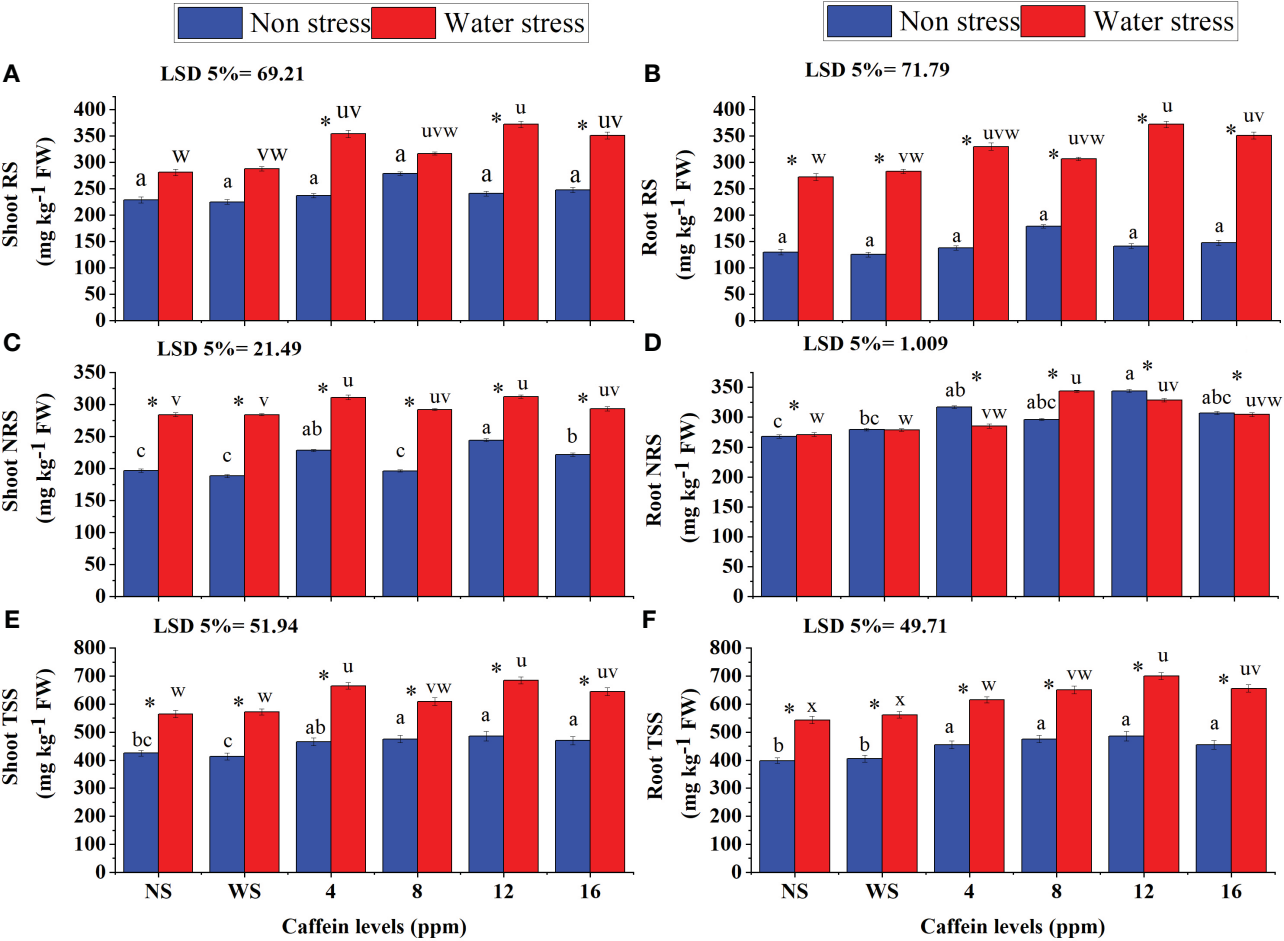
RS **(A, B)**, NRS **(C, D)**, and TSS **(E, F)** in root and shoot, respectively, of wheat seedlings grown from seeds treated with different levels of caffeine under non-stress and PEG-induced water stress (mean ± SE; *n* = 4). Bars with same alphabets do not differ significantly [blue for non-stressed ones (a, b, c, etc.) and red for stressed ones (u, v, w, etc.) against a specific caffeine treatment]. * on bars showing the significant difference between stress (red) and non-stress (blue) in a specific treatment. NS, no soaking; WS, water soaking; RS, reducing sugars; NRS, non-reducing sugars; TSS, total soluble sugars. P values for Shoot RS, Root RS, Shoot NRS, Root NRS, Shoot TSS and Root TSS are P ≤ 0.0029**, P ≤ 0.0000***, P ≤ 0.0000***, P ≤ 0.0311*, P ≤ 0.0000*** and P ≤ 0.0080** respectively

Drought stress significantly increased RS and TSS in both shoots and roots of wheat seedlings. However, NRS content increased only in shoots of seedlings under stress (**Figure 10C**). Seed treatment with caffeine further increased the RS, NRS, and TSS in shoots and roots of wheat seedlings grown under water stress. However, this increment in RS, NRS, and TSS was caffeine dose- and parameter-specific. Regarding RS (**Figures 10A, B**) in roots and shoots under water stress, more increase was found in wheat seedlings raised from seeds pre-treated with 12-ppm level of caffeine, followed by 16- and 4-ppm levels of caffeine. However, under non-stressed conditions, only 8-ppm level was effective in increasing RS. In the case of shoot NRS (**Figure 10C**), 4- and 12-ppm levels of caffeine improved its contents in wheat seedlings grown under both water regimes but for root NRS (**Figure 10D**) 8 and 12ppm were found better under water stress and non stress condition respectively. The highest increase in shoot TSS (**Figures 10E, F**) was found in water-stressed seedlings raised from seeds treated with 4 ppm and 12 ppm caffeine. However, under well-watered conditions, all caffeine doses showed similar effects in improving the shoot TSS in wheat seedlings. Under drought stress, seed treatment with 12 ppm caffeine induced a significant increase in root TSS, followed by 8 ppm and 16 ppm. However, under non-stressed conditions, 8- and 12-ppm levels of caffeine were superior in improving TSS levels in roots of wheat plants.

Growth and germination attributes of wheat seedlings had a strong and positive correlation with the activities of seed Prot, Gluco, and Amy. However, these attributes, including enzyme activities, were negatively correlated with MET and E_50_. MET and E_50_ revealed a strongly positive correlation with each other but had a strongly negative correlation with the activities of germination-related enzymes (Prot, Amy, and Gluco) and growth attributes. Activities of seed hydrolyzing enzymes had a strongly positive correlation with the seed G%, CUE, GE, GI, and growth attributes (**Table 1**).

**Table 1:** Pearson coefficient correlations (*r* ^2^) of seed germination-related attributes, germination enzyme activities, and seedling growth parameters.

	G%	GI	GE	MET	E_50_	CUE	Amy	Prot	Gluco	SL	RL	SFW	SDW	RFW	RDW
G%	1.000														
GI	0.957***	1.000													
GE	0.999***	0.952***	1.000												
MET	−0.973***	−0.963***	−0.970***	1.000											
E_50_	−0.979***	−0.963***	−0.976***	0.999***	1.000										
CUE	0.917***	0.971***	0.916***	−0.916***	−0.912***	1.000									
Amy	0.957***	0.974***	0.952***	−0.962***	−0.960***	0.943***	1.000								
Prot	0.956***	0.978***	0.948***	−0.982***	−0.981***	0.925***	0.985***	1.000							
Gluco	0.937***	0.962***	0.928***	−0.958***	−0.956***	0.918***	0.990***	0.989***	1.000						
SL	0.890***	0.943***	0.880***	−0.958***	−0.951***	0.893***	0.941***	0.971***	0.963***	1.000					
RL	0.859***	0.932***	0.848***	−0.919***	−0.915***	0.870***	0.920***	0.945***	0.939***	0.981***	1.000				
SFW	0.886***	0.939***	0.877***	−0.958***	−0.952***	0.881***	0.937***	0.971***	0.961***	0.999***	0.979***	1.000			
SDW	0.925***	0.975***	0.919***	−0.956***	−0.954***	0.942***	0.947***	0.960***	0.946***	0.971***	0.976***	0.965***	1.000		
RFW	0.866***	0.936***	0.857***	−0.925***	−0.919***	0.884***	0.923***	0.941***	0.937***	0.981***	0.995***	0.977***	0.982***	1.000	
RDW	0.882***	0.952***	0.877***	−0.931***	−0.926***	0.913***	0.925***	0.938***	0.921***	0.965***	0.982***	0.959***	0.991***	0.990***	1.000

G%, germination percentage; GI, germination index; GE, germination energy; MET, mean emergence time; E50, time to 50% germination; CUE, coefficient of uniformity emergence; Amy, amylase; Prot, protease; Gluco, glucosidase; SL, shoot length; RL, root length; SFW, shoot fresh weight; SDW, shoot dry weight; RFW, root fresh weight; RDW, root dry weight.

*** Significant at 0.001 level.

**Figure 11:**
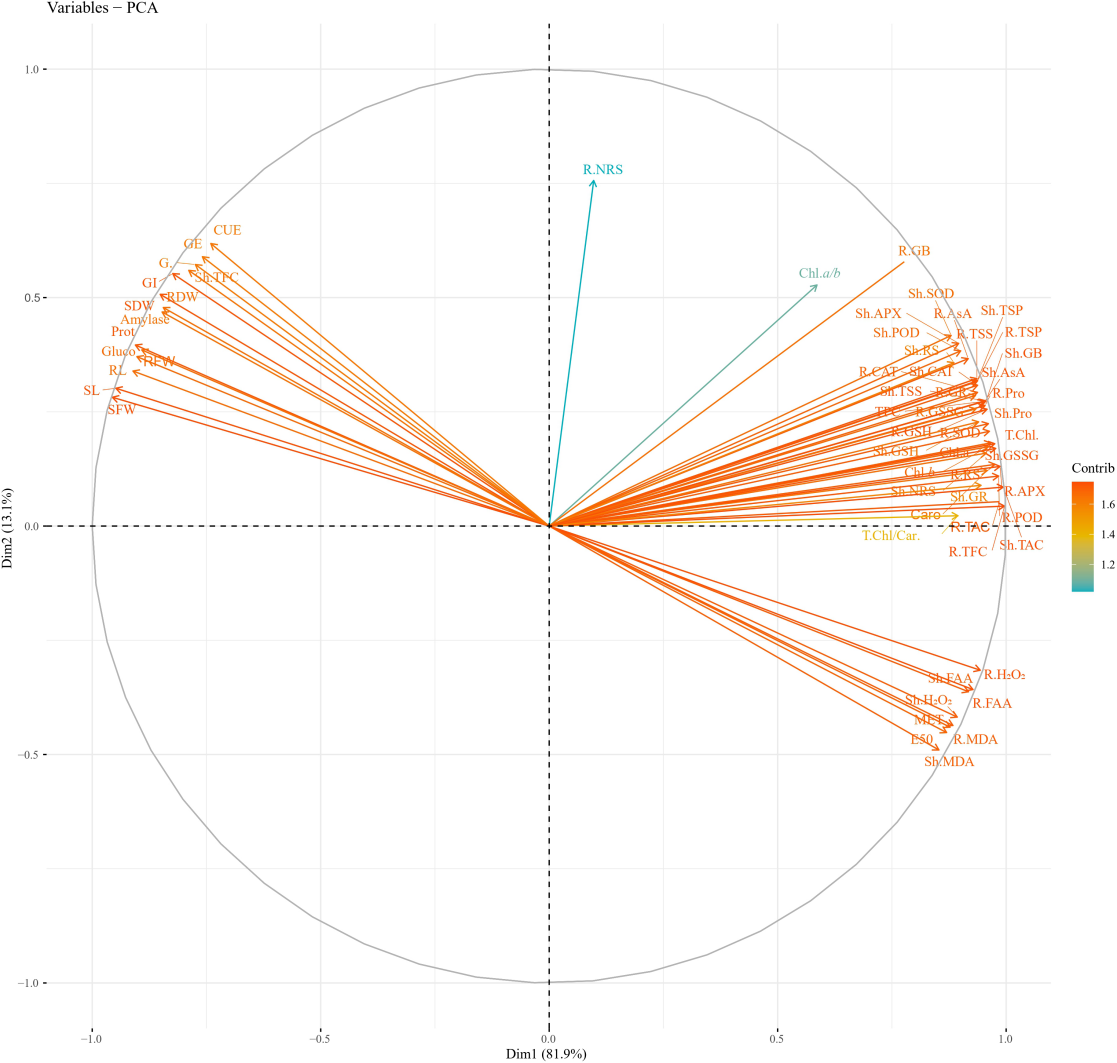
PCA of studied attributes of wheat under non-stress and PEG-induced water stress. PCA, principal component analysis.

**Figure 12:**
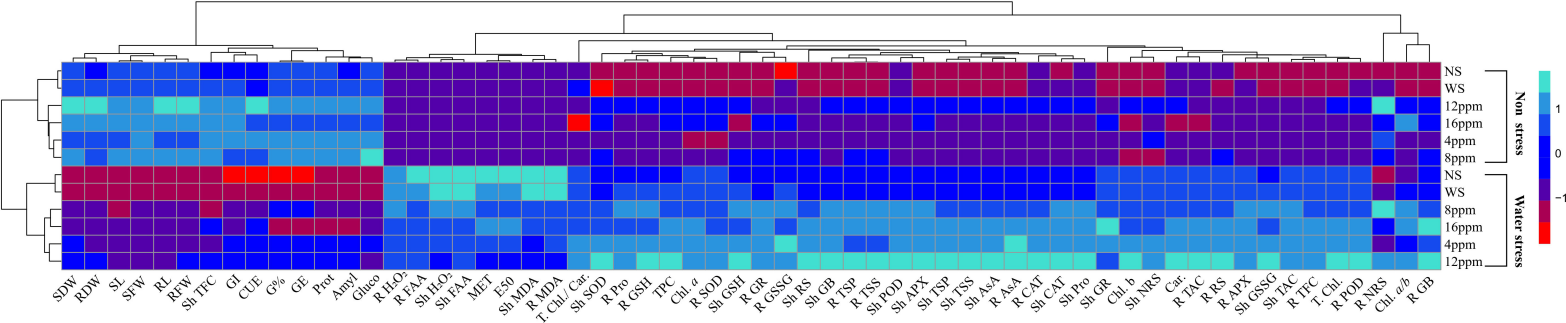
Heatmap analysis of studied attributes of wheat under non-stress and PEG-induced water stress.

PCA values indicated a strong correlation of physio-biochemical attributes with photosynthetic pigments. PCA revealed a positive correlation of growth-related attributes with one another (**Figure 11**; **Table 2**). Root and shoot H_2_O_2_, FAA, and MDA were strongly correlated with one another. Hydrogen peroxide (H_2_O_2_), MDA, and FAA had a strong negative correlation with growth and physio-biochemical attributes. In determining the variance, factor 1 and factor 2 had a major contribution of 81.9% and 13.1%, respectively, with a cumulative value of 95%. Heatmap analysis for studied attributes shows their correlation with the treatments under both water regimes (**Figure 12**). The intensity of color in a column or row against a parameter shows the extent of negative (red color) or positive (green color) correlation with the caffeine doses under both water regimes. The positive effectiveness among the treatments, categorized at the y-axis, reveals that seed priming with 12 ppm caffeine was a standout performer, followed by 4-ppm dose.

**Table 2:** Correlation coefficient values (*r* ^2^) of physiological, biochemical, and growth-related parameters of wheat seedlings when raised from seeds after priming with different caffeine doses under non-stress and water stress imposed by PEG-8000.

	SL	RL	SFW	SDW	RFW	RDW	Chl. *a*	Chl. *b*	Chl. *a*/*b*	T. Chl.	Car.	T. Chl./Car.
Chl. *a*	−0.848***	−0.793***	−0.861***	−0.718***	−0.759***	−0.699***	1.000					
Chl. *b*	−0.858***	−0.824***	−0.872***	−0.730***	−0.804***	−0.727***	0.955***	1.000				
Chl. *a*/*b*	−0.384*	−0.279ns	−0.392*	−0.194ns	−0.242ns	−0.171ns	0.670***	0.604***	1.000			
T. Chl.	−0.862***	−0.817***	−0.876***	−0.732***	−0.789***	−0.720***	0.990***	0.987***	0.646***	1.000		
Car	−0.873***	−0.865***	−0.887***	−0.764***	−0.840***	−0.776***	0.942***	0.973***	0.494**	0.968***	1.000	
T. Chl./Car.	−0.849***	−0.870***	−0.862***	−0.770***	−0.853***	−0.801***	0.842***	0.906***	0.313ns	0.882***	0.970***	1.000
S CAT	−0.802***	−0.760***	−0.815***	−0.644***	−0.735***	−0.655***	0.942***	0.965***	0.688***	0.964***	0.951***	0.880***
R CAT	−0.797***	−0.771***	−0.811***	−0.650***	−0.736***	−0.652***	0.956***	0.973***	0.659***	0.975***	0.969***	0.897***
S POD	−0.747***	−0.707***	−0.761***	−0.576***	−0.679***	−0.597***	0.914***	0.937***	0.702***	0.935***	0.925***	0.853***
R POD	−0.893***	−0.865***	−0.906***	−0.780***	−0.838***	−0.774***	0.978***	0.984***	0.596***	0.992***	0.983***	0.916***
S APX	−0.737***	−0.683***	−0.749***	−0.561***	−0.651***	−0.574***	0.919***	0.913***	0.784***	0.927***	0.889***	0.792***
R APX	−0.901***	−0.856***	−0.911***	−0.775***	−0.835***	−0.779***	0.967***	0.964***	0.657***	0.977***	0.957***	0.886***
S SOD	−0.692***	−0.625***	−0.710***	−0.522**	−0.583***	−0.503**	0.962***	0.923***	0.757***	0.954***	0.878***	0.749***
R SOD	−0.843***	−0.776***	−0.855***	−0.701***	−0.751***	−0.684***	0.992***	0.962***	0.697***	0.989***	0.938***	0.839***
S MDA	−0.953***	−0.927***	−0.949***	−0.969***	−0.934***	−0.938***	0.738***	0.735***	0.268ns	0.745***	0.742***	0.728***
R MDA	−0.964***	−0.931***	−0.963***	−0.965***	−0.935***	−0.936***	0.782***	0.774***	0.313ns	0.787***	0.779***	0.758***
S H_2_O_2_	−0.974***	−0.939***	−0.971***	−0.968***	−0.948***	−0.941***	0.786***	0.788***	0.324ns	0.796***	0.790***	0.769***
R H_2_O_2_	−0.988***	−0.957***	−0.989***	−0.961***	−0.955***	−0.937***	0.859***	0.859***	0.388*	0.869***	0.862***	0.827***
S AsA	−0.823***	−0.791***	−0.837***	−0.679***	−0.758***	−0.685***	0.973***	0.976***	0.659***	0.986***	0.970***	0.889***
R AsA	−0.760***	−0.724***	−0.775***	−0.600***	−0.688***	−0.605***	0.957***	0.957***	0.691***	0.968***	0.940***	0.843***
S TAC	−0.915***	−0.872***	−0.924***	−0.801***	−0.848***	−0.792***	0.983***	0.971***	0.636***	0.989***	0.959***	0.876***
R TAC	−0.920***	−0.909***	−0.929***	−0.823***	−0.882***	−0.827***	0.956***	0.970***	0.544***	0.974***	0.984***	0.936***
TPC	−0.808***	−0.750***	−0.822***	−0.658***	−0.713***	−0.644***	0.989***	0.955***	0.744***	0.984***	0.930***	0.823***
S TFC	0.931***	0.906***	0.925***	0.960***	0.936***	0.950***	−0.646***	−0.665***	−0.135ns	−0.662***	−0.689***	−0.704***
R TFC	−0.928***	−0.888***	−0.938***	−0.824***	−0.865***	−0.813***	0.982***	0.969***	0.592***	0.987***	0.965***	0.895***
S Pro	−0.853***	−0.815***	−0.864***	−0.710***	−0.794***	−0.722***	0.946***	0.968***	0.673***	0.968***	0.959***	0.892***
R Pro	−0.823***	−0.762***	−0.837***	−0.671***	−0.740***	−0.666***	0.977***	0.974***	0.689***	0.987***	0.948***	0.857***
S GB	−0.831***	−0.797***	−0.841***	−0.685***	−0.766***	−0.693***	0.960***	0.971***	0.685***	0.976***	0.957***	0.878***
R GB	−0.555***	−0.498***	−0.573***	−0.360*	−0.445**	−0.371*	0.861***	0.815***	0.834***	0.849***	0.785***	0.667***
S FAA	−0.979***	−0.942***	−0.977***	−0.961***	−0.946***	−0.933***	0.825***	0.825***	0.362*	0.834***	0.820***	0.786***
R FAA	−0.985***	−0.959***	−0.985***	−0.969***	−0.958***	−0.944***	0.837***	0.835***	0.345*	0.846***	0.843***	0.814***
S TSP	−0.789***	−0.758***	−0.805***	−0.641***	−0.716***	−0.648***	0.968***	0.953***	0.696***	0.972***	0.946***	0.856***
R TSP	−0.797***	−0.755***	−0.809***	−0.642***	−0.718***	−0.652***	0.954***	0.940***	0.726***	0.958***	0.930***	0.846***
S TSS	−0.819***	−0.783***	−0.834***	−0.677***	−0.746***	−0.678***	0.982***	0.969***	0.674***	0.987***	0.958***	0.867***
R TSS	−0.794***	−0.752***	−0.806***	−0.639***	−0.715***	−0.649***	0.953***	0.939***	0.728***	0.957***	0.929***	0.845***
S RS	−0.732***	−0.712***	−0.748***	−0.586***	−0.662***	−0.605***	0.937***	0.886***	0.643***	0.924***	0.916***	0.836***
R RS	−0.884***	−0.861***	−0.894***	−0.770***	−0.824***	−0.776***	0.974***	0.943***	0.617***	0.970***	0.963***	0.896***
S NRS	−0.854***	−0.801***	−0.866***	−0.727***	−0.782***	−0.708***	0.958***	0.986***	0.656***	0.982***	0.931***	0.834***
R NRS	0.118ns	0.197ns	0.111ns	0.307ns	0.207ns	0.288ns	0.170ns	0.224ns	0.585***	0.197ns	0.115ns	0.035ns
S GR	−0.866***	−0.813***	−0.877***	−0.758***	−0.786***	−0.752***	0.952***	0.898***	0.676***	0.937***	0.879***	0.773***
R GR	−0.808***	−0.742***	−0.818***	−0.655***	−0.719***	−0.660***	0.955***	0.924***	0.725***	0.951***	0.898***	0.794***
S GSSG	−0.884***	−0.838***	−0.891***	−0.753***	−0.816***	−0.753***	0.967***	0.964***	0.682***	0.977***	0.945***	0.858***
R GSSG	−0.817***	−0.761***	−0.825***	−0.674***	−0.737***	−0.674***	0.962***	0.931***	0.676***	0.958***	0.916***	0.819***
S GSH	−0.826***	−0.786***	−0.838***	−0.692***	−0.759***	−0.694***	0.957***	0.950***	0.579***	0.964***	0.954***	0.890***
R GSH	−0.829***	−0.768***	−0.842***	−0.677***	−0.743***	−0.671***	0.976***	0.971***	0.706***	0.985***	0.945***	0.855***

***, **, and * indicate significance at 0.001, 0.01, and 0.05 levels, respectively.

GE, germination energy; GI, germination index; G%, germination percentage; CUE, coefficient of uniformity emergence; E_50_, time to 50% germination; MET, mean emergence time; Prot, protease; Amy, amylase; Gluco, glucosidase; RDW, root dry weight; RFW, root fresh weight; SDW, shoot dry weight; SFW, shoot fresh weight; RL, root length; SL, shoot length; T. Chl, total chlorophyll; Chl. b, chlorophyll b; Chl. a, chlorophyll a; Chl a/b, chlorophyll ratio; Caro, carotenoids; S TSP, shoot total soluble protein; R TSP, root total soluble protein; R FAA, root free amino acid; S FAA, shoot free amino acid; S TAC, shoot total anthocyanin; R TAC, root total anthocyanin; R TFC, root total flavonoid content; TPC, total phenolic content; S TFC, shoot total flavonoid content; S TSS, shoot total soluble sugar; R TSS, root total soluble sugar; S MDA, shoot malondialdehyde; R MDA, root malondialdehyde; R H_2_O_2_, root hydrogen peroxide; S H_2_O_2,_ shoot hydrogen peroxide; R Pro, root proline; S Pro, shoot proline; R AsA, root ascorbic acid; S AsA, shoot ascorbic acid; S RS, shoot reducing sugars; R RS, root reducing sugars; R NRS, root reducing sugars; S NRS, shoot non-reducing sugars; S GB, shoot glycine betaine; R GB, root glycine betaine; S GSSG, shoot oxidized glutathione; R GSSG, root oxidized glutathione; R GSH, root reduced glutathione; S GSH, shoot reduced glutathione; S SOD, shoot superoxide dismutase; S POD, shoot peroxide dismutase; R SOD, root superoxide dismutase; R POD, root peroxide dismutase; S CAT, shoot catalase; R CAT, root catalase; S CAT, shoot catalase; R APX, root ascorbate peroxidase; S APX, shoot ascorbate peroxidase; R GR, root glutathione reductase; S GR, shoot glutathione reductase.

## 4 Discussion

Maintenance of good crop stand establishment is a pre-requisite for better production, especially in agronomic and field crops. Good crop stand establishment is the function of better seed germination and uniform and speedy seedling emergence. However, failure to achieve good crop stand is seriously disrupting agricultural yields under stressful conditions, especially in low water availability (Batool et al., 2014; Rezayian et al., 2018; Dani and Siswoyo, 2019) in rain-fed environments of semi-arid and arid areas. Soil moisture deficit slows down the pre-germination metabolic activities in seed and hence delays germination and seedling establishment (Basu et al., 2016). The availability of a sufficient amount of water for the imbibition of seeds is a pre-requisite to activate the hydrolyzing enzymes such as Prot, Amy, and Gluco, which break down the large metabolites into smaller ones. These simple metabolites act as building blocks for the newly developing seedlings and are helpful in the fast emergence of seedlings and their early adaptation to the prevailing environment (Perveen et al., 2021). Different ways/techniques, including agronomic practices and seed priming with different chemicals (inorganic or organic) (Ali et al., 2020a; 2020b, Saeed et al., 2023a), are being employed as possible solutions to overcome the problem of poor seedling emergence. Pre-sowing seed treatment performs different functions, depending on the type and nature of applied compounds (Noman et al., 2018b; Ali et al., 2020a; 2020b; Noman et al., 2021). First, it reduces the osmotic potential of the treated seeds, which results in better water absorption for imbibition under low soil water potential, which is necessary for proper activation of the hydrolyzing enzymes. Second, the applied compound directly boosts the metabolic activity of the embryonic region of seeds and expedites cell division and embryo growth in parallel with the help of other metabolic reactions (Ali et al., 2020a). However, the extent of effectiveness depends on the type and concentration of the compound (Noman et al., 2018b; Ali et al., 2020a; Noman et al., 2021).

In the present study, seed priming with caffeine ameliorated the negative effects of drought on different seed germination- and seedling emergence-related attributes of wheat including GE, E_50_, GI, G%, CUE, and mean emergence time. However, better ameliorating effects were observed due to 4- and 12-ppm levels of caffeine than other doses. Good seed germination and speedy seedling emergence are positively associated with better performance of hydrolyzing enzymes necessary for seed germination, such as Prot, Amy, and Gluco. These hydrolyzing enzymes actively take part in providing energy and building blocks to newly developing seedlings by catabolizing seed reserves, such as starch, proteins, and larger sugar molecules (Perveen et al., 2021; Saeed et al., 2023a). In view of the results of the present study, better germination and fast seedling emergence, induced by seed priming treatments, may be the function of caffeine by boosting the activities of Prot, Amy, and Gluco. Previous studies revealed both negative and positive influences of caffeine on the germination of seeds and seedling emergence that were specific to caffeine concentration and plant species. Jeephet (2021) reported that pepper seed priming with 10 mM caffeine showed significant improvements in germination percentage, radicle emergence, germination speed, germination index, seedling vigor, and MET. Moreover, the primed seeds with 20 mM caffeine showed a fast emergence of cotyledons in comparison with non-primed ones (Jeephet, 2021). Further, improvement in seed germination may also be attributed to the role of caffeine in boosting the process of cell division as reported by Sledz et al. (2017). In Bambara groundnut (*V. subterranea*), lower caffeine doses, as a pre-sowing seed treatment, significantly improved the process of seed germination, but with an inhibitory effect at higher doses (Mshelmbula et al., 2018). According to Tunma (2017), caffeine treatment improved seed germination and the proliferation of roots in riceberry plants. The current study also revealed a positive correlation between shoot and root growth with the absorption of caffeine, showing higher content in shoot than root. The uptake of caffeine in the present study after seed priming can be correlated to the earlier findings of Sledz et al. (2017), where it was found that exogenously applied caffeine increased the cell division that was associated with its uptake. The higher caffeine content in shoots may be associated with its translocation from root to shoot (Taiz et al., 2015). More caffeine content in roots and shoots of stressed plants may be due to greater cell density per unit area in water-stressed plants than in non-stressed ones. The increase in translocation of caffeine from roots to shoots and its better retention in stressed plants confirm its beneficial impacts against long-term drought spells. This fact needs to be explored in future studies. Earlier, it was reported that in water-stressed maize, the induction of stress tolerance for better yield by exogenously applied GB was associated with its long-distance translocation and long-term retention in plant parts (Ali and Ashraf, 2011).

The findings of the present study correlate well with studies of Ali et al. (2020), where it was reported that priming of wheat seeds using low doses of the extract of *Cuscuta reflexa* significantly increased the seed germination and seedling emergence-related attributes of wheat under PEG-8000-induced drought stress. The improvement in germination and the seedling establishment was positively correlated with the activity of hydrolyzing enzymes. According to another study, better germination of safflower seeds was the function of the speedy performance of seed hydrolyzing enzymes (Perveen et al., 2021). Another study on safflower and maize concluded that improvement in germination and seedling emergence was associated with high energy states of germinating seeds in the form of bio-photon emission due to the breakdown of large metabolites into smaller ones (Perveen et al., 2022). In the present study, improved germination and seedling emergence in caffeine-treated seeds may be due to enhancement in activities of hydrolyzing enzymes, resulting in energy generation by the breakdown of larger metabolites that were used for various metabolic activities.

In agronomic crops, better growth and biomass of plants are linked with early establishment of seedlings. Early seedling establishment, along with a strong root system, increases the nutrient and water absorption capacity of plants (Ali et al., 2020d). Early seedling establishment in water-deficit environments is of utmost importance for the survival, better biomass production, and yield of plants (Noman et al., 2018b; Ali et al., 2020d). It is well known that longer roots are capable of extracting moisture and nutrients from the soil efficiently and hence are helpful in early seedling establishment under drought conditions (Taiz et al., 2015).

In the present study, seed priming with caffeine significantly reduced the adversities of PEG-8000-induced drought stress on root growth, shoot length, and biomass production, but the extent of amelioration was caffeine dose- and parameter-specific, where 4- and 12-ppm levels were found more effective in increasing the RL and SL than other concentrations. Improvement in root and shoot length was associated with fast germination and early seedling emergence. In previous studies, it was found that seed priming, with different extracts or chemicals, induced speedy germination and early emergence with a strong root system (Ali et al., 2020d; Perveen et al., 2021, Perveen et al., 2022), which is in line with the findings of the current investigation. Boosting cellular metabolic activities and rapid cell division, during germination, results in accelerated growth, with longer roots under water scarcity (Khursheed et al., 2009; Sledz et al., 2017). Seed priming with 10 mM caffeine positively influenced the root and shoot lengths, and growth of newly developed plantlets over control treatment in pepper (Jeephet, 2021). Caffeine application in optimal concentrations increased rooting frequency and accelerated root growth in Logan Thornless blackberry (Muratova et al., 2020). In *Capsicum annuum*, lower doses of caffeine positively influenced the plant height, while inhibitory impacts were found at higher doses (Kumar and Tripathi, 2004). These positive influences of caffeine on root and shoot growth confirm that it may be due to the speedy cellular metabolic activities (Khursheed et al., 2009). It was found that in riceberry, seed pre-treatment with caffeine resulted in better root length and seedling height, which were positively associated with speedy germination (Tunma, 2017).

In the present study, better biomass production and root growth are positively associated with fast germination, induced by caffeine seed treatment. The improvement in growth traits may be the function of better translocation of metabolites during germination and absorption of water and nutrients thereafter, with longer roots. The caffeine-induced enhancement in growth attributes of wheat plants may be the function of stress amelioration by improving metabolic activities. According to previous studies, caffeine treatment improved the growth of sunflower (Khursheed et al., 2009) and pepper (Kumar and Tripathi, 2004) by increasing cellular activities that propelled the plants to better biomass production. It is also well known that caffeine-mediated catabolism yields simple metabolites such as uric acid, allantoin, xanthine, CO_2_, and NH_3_ (Ashihara et al., 2017), which play significant roles in nitrogen assimilation, supportive for better growth and biomass accumulation. Caffeine-mediated catabolism also plays an important role in modulating plant growth under stressful environmental conditions (Watanabe et al., 2010, Watanabe et al., 2014). Moreover, the intermediates of ureide metabolism also support the plants in surviving under stressful environments by playing a role in different metabolic activities. Uric acid, a metabolite produced during caffeine catabolism, was found helpful in reducing the adverse effects of water deficit on the growth of *Arabidopsis* (Kumar et al., 2004). Increment in biomass production of wheat seedlings by caffeine seed treatment is also positively correlated with fast germination, seedling emergence, and longer roots. It shows that in the present study, caffeine catabolism in seed or after its translocation may have played a significant role in ameliorating the adverse impacts of PEG-induced water stress on seed germination process and later on the growth of seedlings. It also confirms the long-term impacts of caffeine at later growth stages because there was more caffeine content in the shoot than in the root, indicating its rapid translocation and potential utility at later growth stages through its catabolism.

Treatment of seeds with low doses of caffeine (4 ppm and 12 ppm) significantly improved the photosynthetic pigments in both stressed and non-stressed wheat seedlings. Improvement in photosynthetic pigments is also positively associated with the increase in biomass production. It is a well-known fact that enhancement in leaf photosynthetic pigments under water stress corresponds well to better stress tolerance (Ali et al., 2020d; Shahid et al., 2022; Shehzad et al., 2022). Therefore, better growth of wheat plants under water deficit may be ascribed to caffeine-mediated improvements in photosynthetic pigments. It is a well-established fact that exogenously applied compounds are absorbed efficiently and translocate to different parts of plants, where they play important roles in regulating different metabolic activities, and the same may be true for seed treatment with caffeine in the present study. Exogenously applied purine alkaloids effectively reduced the adverse impacts of abiotic stresses on photosynthetic pigments, directly or indirectly, by promoting their biosynthesis as reported in *Sedum alfredii* (Chen et al., 2017), *Solanum melongena* (Singh and Prasad, 2015), *Lycopersicon esculentum* (Bali et al., 2018), and *Cucumis sativus* L. (Vitoria and Mazzafera, 1997).

Plants grown from caffeine-treated seeds have also maintained better biosynthesis of cellular metabolites such as sugars, FAA, and TSP, positively correlated with their increased biomass production. These metabolites play important functions in maintaining cellar water balance, necessary for optimum plant growth, by osmotic regulation. The accumulation of these metabolites is also necessary for the plants to tolerate water deficit stress by lowering the osmotic potential of cells, resulting in maintaining the cellular water contents by increased uptake, which is necessary for the continuation of metabolic activities (Shehzad et al., 2022). In the present study, the accumulation of these metabolites, due to pre-sowing seed treatment with caffeine, showed its long-term role in maintaining plant water relations by playing key functions in cellular osmotic adjustment to maintain the cell turgidity, which is necessary for cell division to maintain the growth. Emanuil et al. (2022) reported that in spinach grown under osmotic stress, exogenous application of caffeine played a significant role in maintaining cellular osmotic adjustment for better plant water relations through a significant increase in the accumulations of osmolytes, proteins, and sugars. Moreover, Alkhatib et al. (2016) reported that foliar application of caffeine stimulated the accumulation of sugars in tobacco leaves. Accumulation of sugars improved plant water relations through osmotic adjustment to cope with adverse conditions.

Under deficit irrigation, an increase in lipid peroxidation is a common phenomenon. It triggers the over-production of reactive oxygen species (ROS), which not only damages the cellular membrane but also disturbs other macro-molecules and cellular metabolic processes, including enzyme activities (Saeed et al., 2023a, Saeed et al., 2023b). Increased lipid peroxidation, due to oxidative stress, severely disturbs the biomass production and yield-related attributes of plants. Lipid peroxidation disrupts plant water relations through increased membrane leakage that results in loss of cell turgidity and damage to chloroplastic membranes (Shahid et al., 2022), which causes disturbances in photosynthetic processes and assimilations. In the current study, the accumulation of MDA and H_2_O_2_ in roots and shoots of plants showed an increase in cellular lipid peroxidation. However, seedlings grown from caffeine-treated seeds, especially with 12 ppm caffeine, showed less accumulation of MDA and H_2_O_2_ than untreated ones. It shows that caffeine treatment directly and indirectly reduced lipid peroxidation in plants. Lipid peroxidation is negatively correlated with the dry biomass of wheat seedlings. Caffeine-treated plants are found capable of maintaining better cellular water relations and cellular turgidity for cell division by less leaky membranes. Caffeine also played a role in osmo-regulation in both roots and shoots through the accumulation of osmolytes, resulting in a better ability of plants to uptake water and consequently improve plant growth in terms of dry biomass. Moreover, Sledz et al. (2017) reported that caffeine plays a role in the active mitotic division of cells of apical meristem in broad bean. Second, caffeine-induced reduction in lipid peroxidation is positively associated with better maintenance of chloroplastic membranes and is directly linked with better growth of caffeine-treated plants than non-treated ones. It means that caffeine directly or indirectly played an antioxidative role (Emanuil et al., 2022) in protecting the cellular membranes, including the chloroplastic ones, which is positively correlated with its better translocation from root to shoot.

In the present study, caffeine-induced reduction in lipid peroxidation is also associated with increased activities of enzymatic antioxidants (SOD, POD, APX, and CAT) and accumulation of non-enzymatic antioxidants (AsA, TPC, TFC, TAC, and Car). It indicates an indirect role of caffeine in the antioxidative defense mechanism to reduce lipid peroxidation through ROS scavenging. This antioxidative defense mechanism is also positively associated with the better growth of caffeine-treated plants. It shows that caffeine has a strong indirect impact on maintaining the antioxidative defense mechanism. Improvement in the antioxidative defense mechanism is negatively associated with MDA and H_2_O_2_ levels in roots and shoots. The direct role of caffeine in reducing lipid peroxidation is through its catabolism, which yields a number of metabolites directly involved in reducing the membrane lipid peroxidation by acting as ROS scavengers. It is reported that the exogenous application of caffeine undergoes a purine catabolism pathway that yields the xanthine and allantoin, which have ROS scavenging activity as putative antioxidants and reduce membrane lipid peroxidation (Watanabe et al., 2014; Takagi et al., 2016; Nourimand and Todd, 2017). Moreover, the exogenous application of allantoin reduces the level of reactive oxygen and H_2_O_2_ in leaves of *Arabidopsis thaliana* L., accompanied by an increase in photosynthetic pigment contents (Watanabe et al., 2014; Han et al., 2020). The findings of the present study are in line with those of previous reports, where exogenous application of caffeine decreased the MDA and H_2_O_2_ levels and maintained better photosynthetic pigments, resulting in improved biomass of wheat seedlings. Recently, caffeine-induced modulation in lipid peroxidation, by boosting up the antioxidative defense mechanism, has been reported in osmotically stressed plants of spinach that is linked with better chlorophyll pigments, cellular osmotic adjustment, and better biomass accumulation (Emanuil et al., 2022).

## 5 Conclusions

Caffeine-mediated improvement in growth/biomass of wheat plants is associated with rapid germination and seedling emergence through enhancement in the activities of germination enzymes, maintenance of chlorophyll contents, osmotic adjustment by accumulation of biomolecules, and reduction in ROS production through accumulation of enzymatic and non-enzymatic antioxidants. The enhanced water stress tolerance of wheat seedlings also seems associated with the translocation of caffeine from root to shoot, which correlates well with maintaining the different physio-biochemical mechanisms. Among different levels of caffeine, 12 ppm was found to be the most effective one. The findings of the present study are beneficial for agriculturists working in semi-arid and arid regions to improve wheat production by maintaining better crop stands. In the present study, the role of caffeine in improving germination and seedling establishment is studied on a single wheat genotype under controlled growth conditions. Further studies are recommended in the field under open environmental conditions, on wheat as well as other plant species, for a clear understanding of caffeine’s role in water stress tolerance. Moreover, further studies are also needed to find out the possible economic outcomes in terms of seed yield increments under deficit irrigation conditions.

## Data availability statement

The raw data supporting the conclusions of this article will be made available by the authors, without undue reservation.

## Author contributions

QA: Conceptualization, Formal analysis, Methodology, Project administration, Resources, Supervision, Writing – original draft. RP: Data curation, Formal analysis, Investigation, Methodology, Writing – original draft. FS: Formal analysis, Investigation, Methodology, Visualization, Writing – original draft. HM: Data curation, Formal analysis, Investigation, Methodology, Software, Writing – original draft. SA: Conceptualization, Resources, Software, Writing – review & editing. MI: Formal analysis, Resources, Software, Validation, Writing – review & editing. AA: Conceptualization, Funding acquisition, Resources, Software, Writing – review & editing.
